# Proteomic Alterations in Multiple Myeloma: A Comprehensive Study Using Bone Marrow Interstitial Fluid and Serum Samples

**DOI:** 10.3389/fonc.2020.566804

**Published:** 2021-01-29

**Authors:** Venkatesh Chanukuppa, Ravindra Taware, Khushman Taunk, Tathagat Chatterjee, Sanjeevan Sharma, Venkatesan Somasundaram, Faraz Rashid, Dipankar Malakar, Manas K. Santra, Srikanth Rapole

**Affiliations:** ^1^ Proteomics Lab, National Centre for Cell Science, Pune, India; ^2^ Savitribai Phule Pune University, Pune, India; ^3^ Army Hospital (Research and Referral), New Delhi, India; ^4^ Armed Forces Medical College, Pune, India; ^5^ Sciex, Gurgaon, India; ^6^ Cancer Biology and Epigenetics Lab, National Centre for Cell Science, Pune, India

**Keywords:** multiple myeloma, multipronged quantitative proteomics, bone marrow interstitial fluid, serum biomarkers, SWATH-MS, 2D-DIGE, iTRAQ

## Abstract

Multiple myeloma (MM) is a plasma cell-associated cancer and exists as the second most common hematological malignancy worldwide. Although researchers have been working on MM, a comprehensive quantitative Bone Marrow Interstitial Fluid (BMIF) and serum proteomic analysis from the same patients’ samples is not yet reported. The present study involves the investigation of alterations in the BMIF and serum proteome of MM patients compared to controls using multipronged quantitative proteomic approaches *viz*., 2D-DIGE, iTRAQ, and SWATH-MS. A total of 279 non-redundant statistically significant differentially abundant proteins were identified by the combination of three proteomic approaches in MM BMIF, while in the case of serum 116 such differentially abundant proteins were identified. The biological context of these dysregulated proteins was deciphered using various bioinformatic tools. Verification experiments were performed in a fresh independent cohort of samples using immunoblotting and mass spectrometry based SRM assays. Thorough data evaluation led to the identification of a panel of five proteins *viz*., haptoglobin, kininogen 1, transferrin, and apolipoprotein A1 along with albumin that was validated using ELISA in a larger cohort of serum samples. This panel of proteins could serve as a useful tool in the diagnosis and understanding of the pathophysiology of MM in the future.

## Introduction

Multiple myeloma (MM) accounts for 13% of all hematological malignancies across the world and remains an incurable disease with the worst prognosis (1). The incidence rate of MM varies greatly throughout the world and in developing countries like India, four out of 100,000 individuals are diagnosed with this deadly malignant disease every year (2). MM is characterized by clonal expansion of plasma cells and their subsequent accumulation in the bone marrow which further leads to bone resorption owing to overproduction of antibodies (3). The monoclonal gammopathy of undetermined clinical significance (MGUS) is a benign form of MM that could lead to smoldering myeloma and finally progress into symptomatic myeloma (4). MM is a heterogeneous disease and has several factors attributed to its pathology like hyperdiploidy of specific chromosomes such as 3, 5, 7, 9, 11, 15, 19 and translocations of t(11; 14) (q13; q32) (5). The current clinical practices largely rely on genetic, immunohistochemical and flow cytometric analysis as well as imaging techniques like MRI and PET/CT scan for diagnosis and the evaluation of progression of MM (6). Moreover, researchers also proposed an international staging system for MM using the serum albumin and Beta-2-microglobulin (B2M) levels (7). In addition to these two protein markers, the staging system was further refined with fluorescent *in situ* hybridization technique as well (7, 8).

Interestingly, there is no clinically relevant protein marker yet available for MM diagnosis (6). The current diagnostic methods such as imaging, cytogenetic analysis or immunohistochemistry are expensive, time-consuming, tedious and require highly skilled manpower along with sophisticated equipment. Therefore, there is an unmet need for the biomarkers which can be employed in a clinical setting with high confidence and protein-based biomarkers holds tremendous potential towards it. Moreover, a combination of the routinely used markers such as B2M and serum albumin with newly identified biomarkers panel could be helpful for enhanced specificity and accuracy of MM diagnosis. Mass spectrometry (MS) based proteomics serves as an excellent approach that provides information about protein alterations and modifications in a variety of human samples including the patient’s tissues and body fluids (9). Globally, many research groups have explored and identified potential biomarkers and targets for various cancers using MS-based proteomic approaches (10–14). However, in the case of MM, limited studies have explored the identification of potential candidate markers using proteomic approaches (15–21). Zhang et al. analyzed the pooled serum samples of MM patients towards the identification of markers using liquid chromatography tandem MS/MS (22). In another study, Wang et al. built MM serum diagnostic model and pattern recognition software by using the magnetic bead-based MALDI-TOF MS (23). Lu et al. identified high abundant proteins in MM cells through 2-DE followed by MALDI-TOF MS/MS analysis and the majority of these proteins were categorized under cytoskeletal, chaperone, oxidoreductase and protease class (24). Likewise, Ma and co-authors identified 11 differentially expressed proteins in MM serum using 2-DE followed by MALDI-TOF MS/MS analysis (21). Recently, Bai et al. showed the correlation of fibrinogen alpha chain, dihydropyrimidinase-like 2, platelet factor 4 and alpha-fetoprotein proteins with disease states of MM (20). Apart from that, other proteomic studies reported on MM, so far focused on the identification of proteomic alterations in response to drug treatments (25–29).

To identify the suitable biomarker for MM, the choice of biospecimen for proteomic investigation is very important. Being a proximal biofluid for the hematological malignancies, bone marrow interstitial fluid (BMIF) could serve as a potential source for identifying diagnostic, prognostic and therapeutic markers for various blood cancers including MM (30). On the other hand, blood serum serves as a minimally invasive biofluid and is routinely used in clinical diagnosis. The majority of reported and clinically approved diagnostic markers are serum proteins. Therefore, in this study, we have investigated the proteome alterations in the MM BMIF and serum through multipronged quantitative proteomics by employing two-dimensional difference gel electrophoresis (2D-DIGE), isobaric tags for relative and absolute quantitation (iTRAQ) and Sequential Window Acquisition of all Theoretical Mass Spectra (SWATH-MS) approaches. The statistically significant proteins from both the biological matrices were also compared with each other to identify the common proteins with a similar trend of differential abundance. Additionally, the probable biological and functional roles of the differentially abundant proteins were probed in the manifestation of MM disease pathology by using various *in silico* tools. Furthermore, a selected panel of statistically significant proteins was verified for their differential abundance by immunoblotting as well as MS-based SRM assays. Moreover, potential candidate proteins were also validated in a fresh independent cohort of serum samples using Enzyme-linked immunosorbent assay (ELISA), as it is a minimal invasive fluid that could easily be used for the diagnostics applications. The significance of this study remains in the fact that the proteins which are observed in the BMIF and also reflected in the serum with similar expression profile are proposed as a potential candidate biomarker panel for MM diagnosis. Though BMIF serves as proximal fluid and an excellent source of information on disease pathophysiology, it is an extremely invasive sampling procedure, which puts a lot of stress on the suspected patients of MM. Therefore, the panel of protein markers which are present both in BMIF and serum with a similar expression profile could serve as an excellent alternative and complementary markers with high specificity and accuracy. To the best of our knowledge, this is the first comprehensive proteomics study involving MM clinical bone marrow interstitial fluid and serum samples which utilizes multipronged proteomic approaches.

## Materials and Methods

### Sample Collection

**Figure 1:**
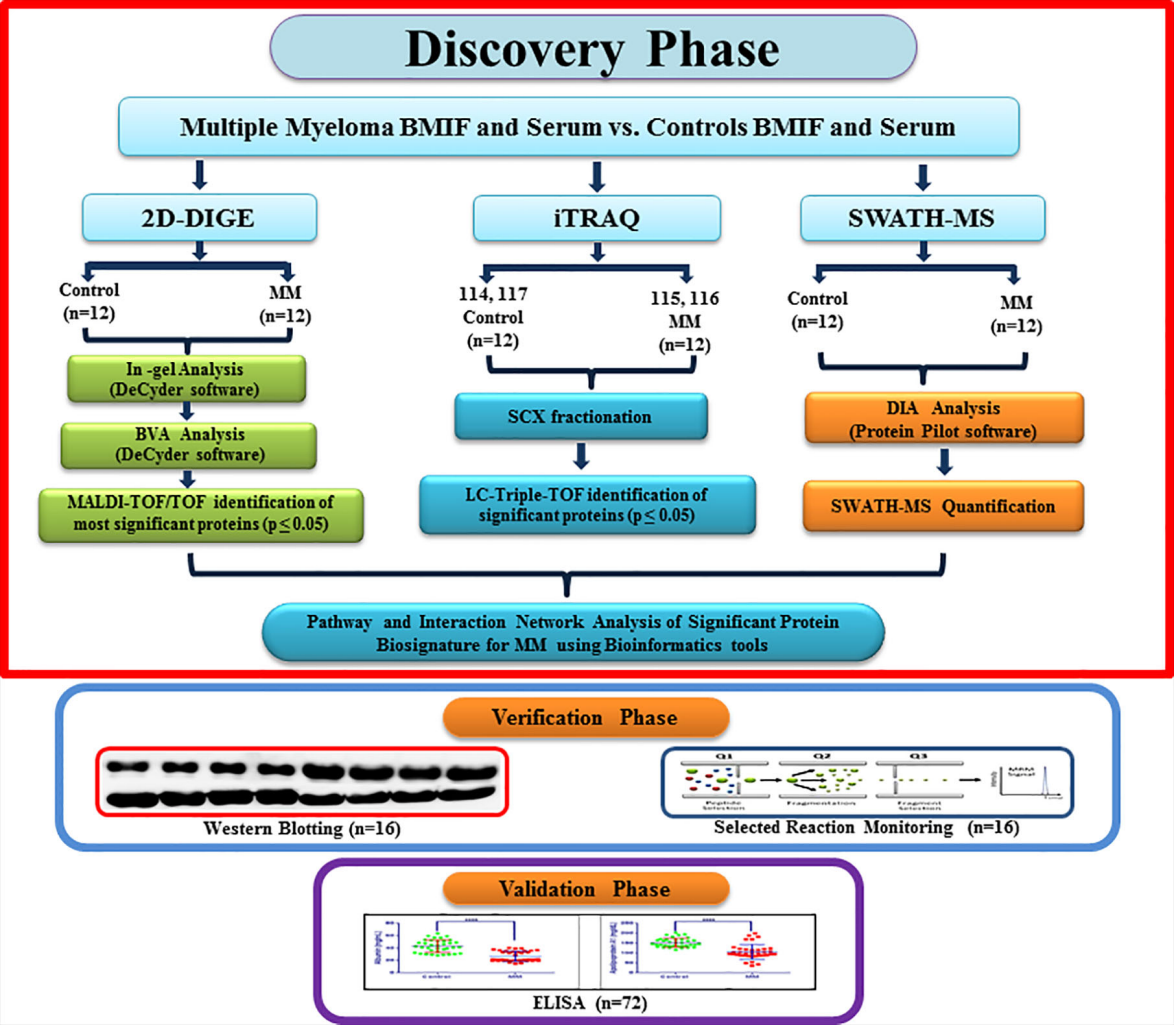
A flowchart depicting experimental design along with the sample allocation strategy for Multiple myeloma BMIF and serum proteomics.

The Institutional Ethics Committee of Armed Forces Medical College, Pune and National Centre for Cell Science, Pune approved this study. A total of 156 volunteers (MM = 64, non-hematological malignancies control = 28, healthy controls = 64) were recruited for the study. Due to the unavailability of bone marrow interstitial fluid from healthy controls, we included the non-hematological malignancy samples in this study as BMIF control samples as these samples are devoid of any hematological malignancies. All the volunteers were informed about the study and written informed consent was obtained from each of them before clinical sample collection. The bone marrow aspirate and peripheral blood samples were simultaneously collected from the same study cohort. Freshly diagnosed naive MM patients without any other malignancy, diabetes and hypertension were recruited for this study. Similarly, controls without any ailments such as diabetes or hypertension were recruited for this study. The average age of the MM subjects recruited for this study was 63.95 years and that of healthy volunteers and non-hematological malignancy patients was 57.54 and 56.2 years, respectively (**Table S1**). Parameters such as serum creatinine and M protein concentrations were considered for discriminating the early stage of MM samples. Total 24 BMIF and 24 serum samples (MM = 12, control = 12) were utilized for the discovery phase. For 2D-DIGE and iTRAQ, 3 pooled samples were used for each set and 4 sets of experiments were performed. Whereas 12 individual samples were used for SWATH-MS experiments and each sample was acquired in triplicate. A separate cohort of samples of MM patients (n = 16) and respective controls (n = 16) was used for WB and SRM-based verification assays. The verification experiments included a different set of 8 controls and 8 MM samples for all the western blotting experiments. Similarly, the SRM experiments comprised of 8 biological replicates from the different verification sample set. Finally, in the validation phase, we performed ELISA experiments on serum samples obtained from a separate cohort of 72 volunteers (MM = 36, control = 36). A flow chart depicting the design of the experiment and sample allotment strategy is shown in **Figure 1**.

### Protein Extraction and Sample Preparation

The bone marrow aspirate and blood samples were incubated for 30 min at room temperature and then centrifuged at 3,000 rpm for 10 min at 10°C to separate the BMIF and serum respectively. The BMIF and serum samples were transferred in sterile cryovials, labeled and stored at −80°C freezer until further experiments. BMIF and serum samples were depleted for most abundant proteins such as albumin and IgG by using affinity chromatography based spin trap format depletion kit and desalted using a 2D clean-up kit (both GE Healthcare, USA) as per instructions provided by the manufacturer. BMIF and serum protein pellets, thus obtained, were dissolved in rehydration buffer (7 M Urea, 2 M Thiourea and 2% CHAPS) and the protein estimation was performed by a 2D Quant kit (GE Healthcare, USA).

### Cy Dye Labeling and 2D-DIGE Analysis

The 2D-DIGE experiments were performed as per the protocol reported previously by Rapole and co-authors (31). Briefly, 60 µg of BMIF and serum proteins from controls and MM patients were labeled with 400 pmol of CyDyes Cy5, Cy3 respectively. Moreover, an internal control having 1:1 concentration of both samples was constituted and labeled with Cy2. The labeled samples were rehydrated on a 24 cm linear IPG strip (pH 4–7), and the strips were subjected to isoelectric focusing (IEF) through EttanIPGphor 3 instrument (GE Healthcare, USA). Following the first dimension IEF procedure, the second-dimension SDS-PAGE was performed and the gels were scanned through the Typhoon FLA 9500 biomolecular imager (GE Healthcare, USA). The images were analyzed using DeCyder 2D software; version 7.0 (GE Healthcare, USA) for both the differential in-gel analysis and biological variation analysis (BVA). The statistically significant protein spots passing the t-test (p-value ≤ 0.05) and present in all the gels were selected for MALDI-TOF/TOF identification.

### In-Gel Digestion and Protein Identification Using MALDI-TOF/TOF

Differentially expressed protein spots identified in 2D-DIGE were excised automatically through Ettan spot picker (GE Healthcare, USA) from the preparative gel and subjected to in-gel digestion of the proteins as reported earlier (32). Briefly, the gel pieces were reduced with DTT and alkylated with IAA and subjected to enzymatic digestion using trypsin (100 ng) by keeping at overnight incubation at 37°C. The tryptic peptides from in-gel digestion were extracted and spotted on a MALDI plate with CHCA matrix (10 mg/ml). Protein identification was performed using a 4,800 MALDI-TOF/TOF mass spectrometer (AB Sciex, USA) linked to a 4,000 series explorer software (v.3.5.3) equipped with Nd: YAG 355 nm laser and a repetition rate of 200. The mass range of 800 to 4,000 Da was used, and the mass spectra were acquired in reflector mode with 20 kV and 18 kV as acceleration and extraction voltages, respectively. MS/MS spectra were acquired for the 12 most abundant precursor ions, with a total accumulation of 2,500 laser shots and collision energy of 2 kV. The MASCOT version 2.1 (http://www.matrixscience.com) was used for the data analysis by keeping the taxonomy as *Homo sapiens*, database as SwissProt, enzyme as trypsin, oxidation of methionine as variable and carbamidomethylation of cysteine as a fixed modification. The MS mass tolerance was set as 75 ppm and MS/MS as 0.4 Da.

### iTRAQ Labeling and Prefractionation

Hundred microgram protein from MM BMIF and serum [three samples pooled (one set), total four sets] and respective controls [three samples pooled (one set), total four sets] were subjected to in-solution digestion using trypsin. The four plex iTRAQ labeling of digested peptides was performed as per the manufacturer’s protocol (SCIEX, USA). Briefly, iTRAQ reagents dissolved in ethanol were added to the respective protein sample i.e., 114-controls, 115-MM samples, 116-MM samples and 117-controls and incubated at RT for 1 h. The labeled samples were pooled and concentrated using SpeedVac (Savant- SPD 1010, Thermo Electron Corporation, USA). Labeled peptide sample was fractionated by SCX chromatography (Poly-SULFOETHYL A column, 100 × 4.6 mm, 5 mm, 300 Å, PolyLC, Columbia, MD) using a Shimadzu HPLC. The fractionated peptide samples were again concentrated under vacuum and desalted using C18 ZipTips (Millipore, USA) before performing the LC-MS/MS analysis. The desalted peptide samples were separated and analyzed through Eksigent MicroLC 200 System (Eksigent, Canada) which was coupled to a Triple TOF 5600 high-resolution mass spectrometer (SCIEX, USA). The peptides were separated using a linear gradient of 7–35% ACN for 60 min for peptide elution from the analytical column with a flow rate of 8 μl/min. The column temperature was set as 40°C, and the autosampler temperature was kept as 4°C. The mass spectrometric analysis was executed with m/z ranging from 100–3,200, MS scan rate of six spectra, MS/MS scan rate of three spectra, with top 15 peaks for MS/MS analysis. The protein identification and quantitation were performed by ProteinPilot software (version 4.0, SCIEX) through the SwissProt database with 1% FDR and one missed cleavage as input parameters.

### SWATH-MS Analysis

The label-free SWATH-MS analysis was performed on BMIF and serum samples (12 MM and 12 controls), which were depleted for Albumin and IgG. An equal amount of BMIF and serum proteins were subjected to trypsin digestion, and the peptides were analyzed using a Triple TOF 5600 mass spectrometer (SCIEX, USA) equipped with Eksigent Nano 2D Ultra 2D plus (Eksigent, Canada) having an Eksigent Nano LC 3C18 CL reverse phase column (75 µm × 15 mm, 3 µm, 120 Å) along with NanoLC Trap Chrom XP C-18-CL (3 µm, 120 Å, 350 µm × 0.5 mm) column. Data dependent analysis (DDA) was performed for the individual samples to generate high quality spectral ion libraries for SWATH-MS analysis, by operating the mass spectrometer with specific parameters as mentioned elsewhere (33). Technical triplicates of SWATH-MS were carried out for each BMIF and serum samples. For SWATH-MS experiments, the instrument was tuned to optimize the quadrupole settings for the selection of the precursor ion window of 25 m/z width. An isolation width of 26 m/z with 1 m/z for the window overlap was used and a set of 34 overlapping windows was built for covering the precursor mass range of 400–1,250 m/z. The SWATH-MS/MS spectra were derived from 100 m/z to 2,000 m/z. The ions were fragmented in the collision cell using rolling collision energy (CE) with an additional CE spread of ±15 eV. A dwell time of 96 ms was used for all fragment-ion scans in high-sensitivity mode, and for each SWATH-MS cycle, a survey scan in high-resolution mode was acquired for 100 ms, resulting in a duty cycle of 3.33 s. The dual-source parameters were as follows: ion source gases GS1, GS2, curtain gas at 25 psi, temperature 200°C, and ion spray voltage floating at 5,500 V. High-quality spectral ion libraries were generated for SWATH analysis through DDA of individual samples. The peak extraction and spectral alignment were performed using the Peakview software (version 2.2, SCIEX, USA) with the following specific parameters: no. of peptides two, no. of transitions 10, peptide confidence 99%, XIC width 30 ppm, XIC extraction window 3 min. The data was further subjected to MarkerView software (version 1.3.1, SCIEX, USA) to get statistical data interpretation. In MarkerView, the results were shown as three output files containing AUC of the ions, the summed intensity of peptides for protein and the summed intensity of ions for the peptide. The summed intensity of peptides was used for the further relative quantitative and multivariate statistical analysis using MetaboAnalyst 3.0 and SIMCA 14.1 platforms. The statistical significance was tested using the student’s t-test and FDR corrections. The reproducibility of the data was checked using retention time matching of some of the selected peptide spectra. The data obtained from the proteomic analysis were subjected to mathematical normalization in order to obtain the Gaussian distribution. The data were normalized to constant sum, cube root transformed and auto-scaled using MetaboAnalyst web application.

### Functional and Interaction Analysis Using Bioinformatics Tools

Proteins that were significantly differentially expressed (p-value < 0.05 and FC ≥ 1.5/≤ 0.67) were interpreted using Database for Annotation, Visualization and Integrated Discovery (DAVID version 6.8, http://david.abcc.ncifcrf.gov/home.jsp), Protein Analysis Through Evolutionary Relationships (PANTHER version 11.1, http://www.pantherdb.org/) analysis and Ingenuity Pathway Analysis (IPA). DAVID analysis was performed by selecting functional annotation, Uniprot accession as identifier ID and extracted the biological functions. Similarly, PANTHER analysis was performed by keeping the *Homo sapiens* as a selected organism. Functional classification viewed in graphic charts for select analysis and exported the molecular function, biological process, cellular component, protein class and pathways. Likewise, *in silico* bioinformatics analysis was performed by Ingenuity Pathway Analysis (IPA) software (Qiagen Bioinformatics, India) to identify the Canonical pathways, upstream regulators and toxicology functions.

### Western Blot Based Verification

Western blot analyses of BMIF and serum samples (MM = 8, Control = 8) were performed as described previously (34). Briefly, BMIF and serum proteins were separated on a 12% SDS PAGE gel (40 µg per well) and then transferred onto a nitrocellulose membrane under semi-dry conditions using ECL semi-dry transfer unit (GE Healthcare, USA). Western blot was performed with a monoclonal/polyclonal antibody against Haptoglobin, Kininogen1, Alpha 1 antitrypsin, Gelsolin, Zinc-2-alpha glycoprotein, Apolipoprotein A1, Transferrin, *β*-actin (all from Sigma Aldrich, USA). An appropriate secondary antibody conjugated with horseradish peroxidase enzyme (HRP) (GE Healthcare, USA) was employed to detect the respective primary antibody. After treating with a chemiluminescent substrate, the blots were visualized by ImageQuant LAS 4000 instrument (GE Healthcare, USA).

### Selected Reaction Monitoring Based Verification

The differentially expressed BMIF and serum proteins were further cross-verified by SRM assays using a new cohort of samples (MM = 8, control = 8). Transitions for each of the proteins to be verified through the SRM experiments were established *via in silico* approach on SKYLINE 3.1 software, cross-referred from SRM Atlas, MRM based public libraries and literature (33, 35, 36). Tryptic peptides with an average length of 5–10 amino acids, devoid of residues prone to post-translational modifications were chosen for establishing SRM transitions. Unique peptides for each protein were taken as a target and the collision energy obtained from SKYLINE software was used. The unique peptide transition list was imported to 4000 Q-TRAP LC-MS/MS system (SCIEX, USA) attached to an Eksigent Micro LC System. SB C18 micro LC column (0.3 mm × 100 mm, 300 Å pore size, 5 µm particle size) from Agilent was used for the separation of peptides with 6 µl flow rate. The raw data files were analyzed using Skyline software for further quantitation. All the spectra were manually interpreted for their differential expression.

### Enzyme-Linked Immunosorbent Assay Based Validation

In the validation phase, we used a separate cohort of 72 volunteers (MM = 36, control = 36) to perform ELISA based validation in serum samples. All the ELISA experiments were performed according to the manufacturer’s instructions. We validated a panel of five proteins with the Human Haptoglobin Quantikine ELISA kit (catalog #DHAPG0; R&D Systems, Inc., Minneapolis, USA), Human Kininogen DuoSet ELISA kit (catalog #DY1569-05; R&D Systems, Inc., Minneapolis, USA), Human Apolipoprotein A-I Quantikine ELISA Kit (catalog #DAPA10; R&D Systems, Inc., Minneapolis, USA), Human sTfR Quantikine IVD ELISA Kit (1 Kit) (catalog #DTFR1; R&D Systems, Inc., Minneapolis, USA), Human Serum Albumin DuoSet ELISA, 15 Plate (1 KIT) (catalog #DY1455; R&D Systems, Inc., Minneapolis, USA). The optical density of the each sample was determined by using an EPOCH 2 microplate reader (Biotek, USA) set to 450 nm and 570 nm. Optical imperfections correction was done by subtracting the readings of 570 nm from 450 nm readings. Further quantitative analysis was performed using GraphPad Prism software.

## Results

### Proteome Alterations in MM BMIF and Serum Using Multipronged Proteomic Approaches

To identify the MM induced differentially expressed proteins in MM BMIF and serum samples, the multipronged quantitative proteomic approaches were employed to maximize the proteomic coverage. The proteins identified in both the biomatrices with similar differential regulation and statistical significance were projected as potential candidate biomarkers. The results obtained from the experiments are discussed henceforth.

#### Identification of BMIF and Serum Proteome Alterations in MM Using 2D-DIGE

**Figure 2:**
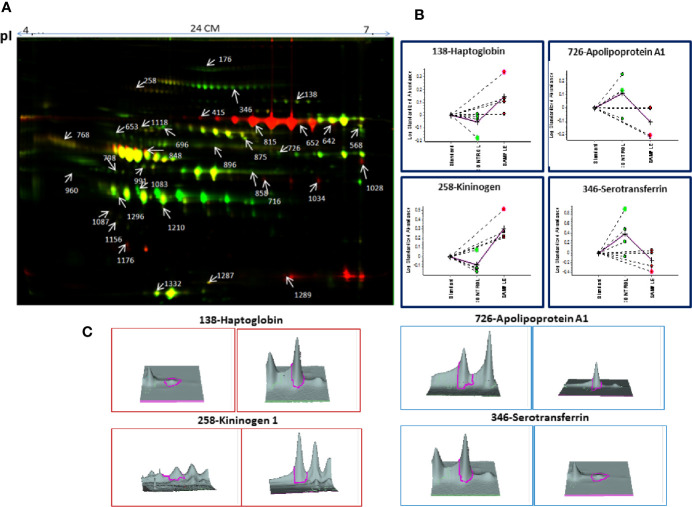
**(A)** The representative 2D-DIGE image for MM BMIF *vs.* Control BMIF. **(B)** Dysregulation profile for some of the identified proteins obtained through BVA module of DeCyder software and **(C)** 3-D View of these proteins obtained through BVA module of DeCyder software.

Decyder software revealed 1,450–1,600 protein spots which emerged as differentially expressed proteins in MM BMIF as compared to control. Biological variations of the gels were compared against a master gel through the BVA module to obtain statistically significant protein spots. Finally, 186 protein spots showed the statistical significance of *p-*value ≤0.05 (student’s t-test) with ≥1.5 fold change for up-regulation and ≤0.67 as fold change for down-regulation. Out of these differentially expressed protein spots, 96 protein spots were up-regulated and 90 protein spots showed down-regulation as compared to control. A total of 112 protein spots were identified through the 4800 MALDI-TOF/TOF MS (Sciex), and 31 proteins were found to be non-redundant. Of these 31 non-redundant proteins, 19 proteins were found to be up-regulated, while 12 proteins showed decreased expression in MM as compared to control. The list of differentially expressed BMIF proteins identified using 2D-DIGE is mentioned in **Table S2**. A representative combined 2D-DIGE image with BVA views showing 3D differential expression of the selected protein spots is depicted in **Figure 2**.

**Figure 3:**
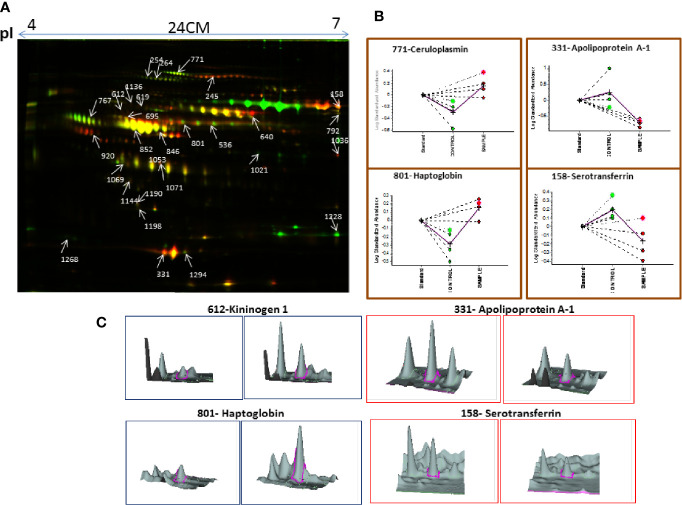
**(A)** The representative 2D-DIGE image for MM serum *vs.* Control serum. **(B)** Dysregulation profile for some of the identified proteins obtained through BVA module of DeCyder software and **(C)** 3-D View of these proteins obtained through BVA module of DeCyder software.

Similarly, serum 2D-DIGE analysis revealed approximately 1,200–1,300 protein spots out of which 153 protein spots showed differential regulation in MM with criteria of statistical significance of *p-*value ≤0.05 and fold change ≥1.5/≤0.67. Out of these differentially expressed protein spots, 90 protein spots were up-regulated and 63 protein spots showed down-regulation. A total of 96 proteins were identified through the MALDI-TOF/TOF MS, and 29 proteins were found to be non-redundant. Of these non-redundant proteins, 18 proteins were up-regulated and 11 proteins were down-regulated in MM as compared to control. The list of differentially expressed serum proteins identified using 2D-DIGE is mentioned in **Table S3**. A representative combined 2D-DIGE image with BVA views showing 3D differential expression of the selected protein spots is depicted in **Figure 3**.

#### Identification of BMIF and Serum Proteome Alterations in MM Using iTRAQ

A total of 719 proteins were identified in BMIF using iTRAQ and out of these 183 proteins were found as differentially expressed with criteria of fold change ≥1.5/≤0.67. A total of 91 proteins showed up-regulation and 92 proteins showed down-regulation in MM BMIF as compared to the control samples. The complete list of significant differentially regulated BMIF proteins identified using iTRAQ methodology is provided in **Table S4**.

In serum, a total of 650 proteins were identified using iTRAQ and out of these, 68 proteins were found as differentially expressed based on the fold change criteria of ≥1.5/≤0.67. Among differentially expressed proteins, 30 proteins were up-regulated and 38 showed down-regulation in MM patients as compared to healthy controls. The complete list of significant differentially regulated serum proteins identified using iTRAQ methodology is provided in **Table S5**.

#### Identification of BMIF and Serum Proteome Alterations in MM Using SWATH-MS

**Figure 4:**
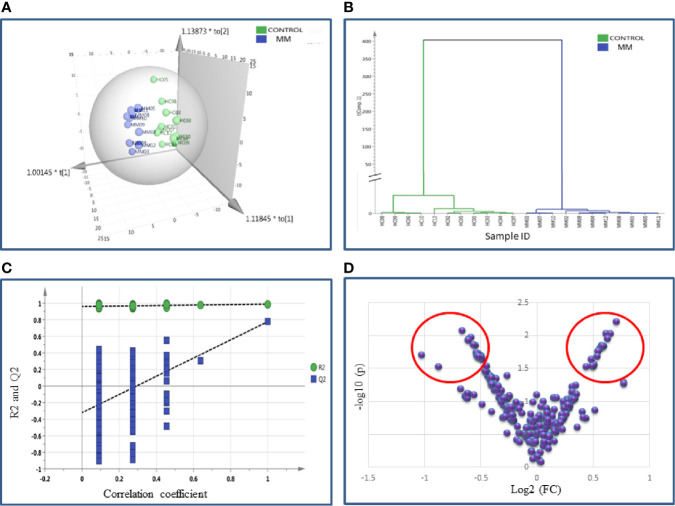
Multivariate statistical analysis of MM BMIF *vs.* Control dataset using SWATH MS data. **(A)** OPLS-DA score plot for MM *vs.* Controls. **(B)** Dendrogram showing the hierarchical clustering between MM and controls based on DEPs. **(C)** Permutation test statistics of the OPLS-DA model of MM *vs.* Controls with Y-axis intercepts: R2 = (0.0, 0.894), Q2 = (0.0, −0.426), **(D)** V-Plot showing the most significant proteins in red circles. One sample in MM was removed from the SIMCA analysis as they had high degree of variation hence the data shown is for eleven MM samples and twelve controls.

SWATH-MS library was built for 1236 BMIF proteins and out of these 283 proteins were commonly identified in all samples. The statistical analysis revealed 104 proteins differentially expressed out of which 41 proteins showed a pattern of up-regulation and 63 proteins showed down-regulation in MM samples as compared to controls (**Table S6**). The differential expression threshold criteria were the same as mentioned in section *Identification of BMIF and Serum Proteome Alterations in MM Using 2D-DIGE* and *Identification of BMIF and Serum Proteome Alterations in MM Using iTRAQ*. Multivariate statistical analysis such as OPLS-DA for BMIF SWATH-MS data revealed good discrimination between the control and the MM groups (**Figure 4**).

**Figure 5:**
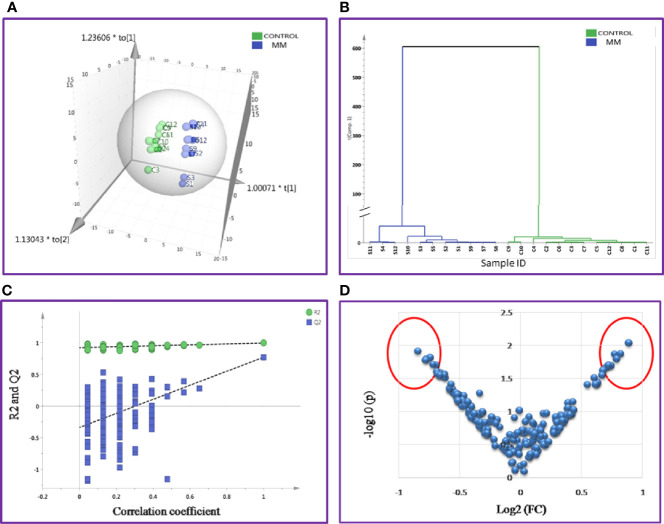
Multivariate statistical analysis of MM serum *vs.* Control dataset using SWATH MS data. **(A)** OPLS-DA score plot for MM *vs.* Controls. **(B)** Dendrogram showing the hierarchical clustering between MM and controls based on DEPs. **(C)** Permutation test statistics of the OPLS-DA model of MM *vs.* Controls with Y-axis intercepts: R2 = (0.0, 0.924), Q2 = (0.0, −0.338), **(D)** V-Plot showing the most significant proteins in red circles. One sample in MM was removed from the SIMCA analysis as they had high degree of variation; hence the data shown is for eleven MM samples and twelve controls.

Likewise, the SWATH-MS library was also built for a total of 1,184 serum proteins and out of these, 213 proteins were consistently observed in all samples. A total of 42 differentially expressed proteins were identified and out of which 18 proteins were up-regulated and 24 proteins showed down-regulation in MM samples as compared to controls (**Table S7**). The differential expression threshold criteria applied were the same as mentioned above. Furthermore, multivariate statistical analysis such as OPLS-DA revealed distinct clustering of MM and healthy control serum samples in score plots (**Figure 5**).

#### Identification of BMIF and Serum Proteome Alterations Using All Three Approaches

**Figure 6:**
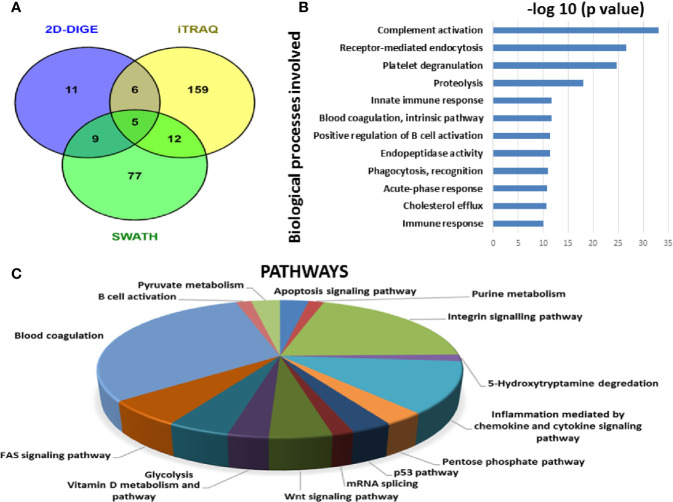
Bioinformatic analysis of the BMIF differentially regulated protein dataset. **(A)** Representative Venn diagram for the differentially regulated proteins found through all three proteomic approaches used for bioinformatics analysis. **(B)** Biological process of the differentially regulated proteins using DAVID and **(C)** Pathways altered in the MM BMIF vs. Control BMIF dataset using PANTHER.

Since each of the quantitative proteomic approaches has its advantages and limitations, combining all these approaches in the discovery phase results in a maximum number of differentially expressed proteins. Using multipronged proteomic approaches, a total of 279 non-redundant BMIF proteins were found to be differentially regulated in MM (**Table S8**). Overall, among all of these differentially expressed proteins, 11 proteins were identified in both 2D-DIGE and iTRAQ, 17 proteins were observed in both iTRAQ and SWATH-MS, 14 proteins were common in both SWATH-MS and 2D-DIGE, and five proteins were presented in all the three approaches (**Figure 6A**). Interestingly, 11 proteins were observed only in DIGE, 159 proteins were specifically observed using the only iTRAQ, and 77 proteins were observed using only the SWATH-MS approach. The partial list of statistically significant differentially regulated proteins is summarized in **Table 1**.

**Table 1:** Partial list of differentially regulated BMIF proteins identified in MM.

Sr. No	UniProt ID	Protein Name	FC in DIGE	FC in iTRAQ	FC in SWATH	Molecular function	Biological process
1	P00738	Haptoglobin	1.78	1.51	1.55	antioxidant activity	Acute-phase response
2	P01857	Ig gamma-1 chain C region	2.49	5.84	0.45	antigen binding	B cell receptor signaling pathway
3	P01591	Ig J chain	1.69	5.70	20.27	antigen binding	adaptive immune response
4	P01871	Ig mu chain C region	0.28	6.65	15.71	antigen binding	adaptive immune response
5	P0DJI8	Serum amyloid A protein	2.38	1.89	—	chemoattractant activity	activation of MAPK activity
6	P01834	Ig kappa chain C region	—	9.49	4.98	antigen binding	B cell receptor signaling pathway
7	P01767	Ig heavy chain V-III region	—	3.13	2.34	antigen binding	complement activation
8	P00450	Ceruloplasmin	—	2.52	2.33	chaperone binding	cellular iron ion homeostasis
9	P00747	Plasminogen	1.57	—	2.29	apolipoprotein binding	blood coagulation
10	P04004	Vitronectin	2.25	—	1.63	collagen binding	cell adhesion
11	P01009	Alpha-1-antitrypsin	1.95	4.23	—	identical protein binding	acute-phase response
12	P01011	Alpha-1-antichymotrypsin	2.1	—	1.96	DNA binding	acute-phase response
13	P25311	zinc-alpha-2-glycoprotein	1.83	2.38	—	ribonuclease activity	transmembrane transport
14	P01042	Kininogen	3.94	—	2.82	endopeptidase inhibitor activity	blood coagulation, intrinsic pathway
15	P02768	Serum albumin	0.23	0.52	0.52	antioxidant activity	cellular protein metabolic process
16	P02675	Fibrinogen beta chain	0.34	0.36	—	chaperone binding	adaptive immune response
17	P80748	Ig lambda chain V-III region	—	0.67	0.54	antigen binding	complement activation
18	P01024	Complement C3		0.60	0.66	endopeptidase inhibitor activity	inflammatory response
19	P05452	Tetranectin	—	0.55	0.47	calcium ion binding	bone mineralization
20	P02786	Transferrin receptor protein 1	—	0.22	0.17	double-stranded RNA binding	cellular iron ion homeostasis
21	P02774	Vitamin D binding protein	0.25	—	0.64	actin binding	vitamin D metabolic process
22	P02787	Serotransferrin	0.38	—	0.42	ferric iron binding	actin filament organization
23	P02647	Apolipoprotein A-1	0.23	0.21	—	amyloid-beta binding	cellular protein metabolic process
24	P06396	Gelsolin	0.61	—	0.48	actin binding	actin filament capping

**Figure 7:**
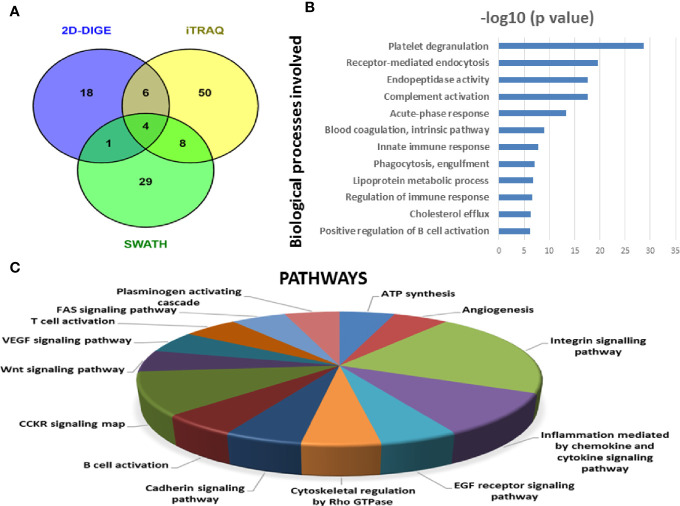
Bioinformatic analysis of the differentially regulated protein dataset. **(A)** Representative Venn diagram for the differentially regulated proteins found through all three proteomic approaches used for bioinformatics analysis. **(B)** Biological process of the differentially regulated proteins using DAVID and **(C)** Pathways altered in the MM serum vs. Control serum dataset using PANTHER.

Similarly, by using multipronged proteomic approaches, 116 non-redundant serum proteins were found to be differentially regulated in MM (**Table S9**). Out of the 116 proteins, 10 proteins were common in both 2D-DIGE and iTRAQ, 12 proteins were detected in both iTRAQ and SWATH-MS, five proteins were identified in both SWATH-MS and 2D-DIGE, and four proteins were observed in all the three approaches (**Figure 7A**). Eighteen proteins were observed only in DIGE, 50 proteins were specifically detected using only iTRAQ, and 29 proteins were identified using only SWATH-MS approach. The partial list of statistically significant and differentially regulated proteins is illustrated in **Table 2**. Identification of proteins in two or more approaches provides its definitive association with a particular disease, in this case MM.

**Table 2:** Partial list of differentially regulated serum proteins identified in MM.

Sr. No	UniProt ID	Protein Name	FC in DIGE	FC in iTRAQ	FC in SWATH	Molecular function	Biological process
1	P00738	Haptoglobin	1.66	1.76	2.22	Antioxidant activity	Acute-phase response
2	P01011	Alpha-1-antichymotrypsin	2.1	1.56	1.95	DNA binding	Acute-phase response
3	P01042	Kininogen 1	2.87	—	—	Cysteine-type endopeptidase	Cellular protein metabolic process
4	P0DJI8	Serum amyloid A-1 protein	—	1.95	8.25	Chemoattractant activity	Activation of mapk activity
5	P25311	Zinc-alpha-2-glycoprotein	1.83	—	—	Glycoprotein binding	Cell adhesion
6	P01009	Alpha-1-antitrypsin	1.95	—	—	Glycoprotein binding	Acute-phase response
7	P00450	Ceruloplasmin	2.16	—	—	Chaperone binding	Iron ion homeostasis
8	P02763	Alpha-1-acid glycoprotein 1	—	1.63	2.27	—	Acute-phase response
9	Q06033	Inter-alpha-trypsin inhibitor heavy chain H3	—	1.88	1.97	Endopeptidase inhibitor activity	Hyaluronanmetabolic process
10	P01591	Ig J chain	4.38	3.30	1.66	Antigen binding	Immune response
11	P61769	Beta-2-microglobulin	—	1.73	—	Glycoprotein binding	Antibacterial humoral response
12	P43652	Afamin	—	0.38	0.61	Vitamin E binding	Vitamin transport
13	P43251	Biotinidase	—	0.38	0.57	Biotinidase activity	Biotin metabolic process
14	P29622	Kallistatin	—	0.30	0.54	Serine-type endopeptidase	Platelet degranulation
15	P05452	Tetranectin		0.51	0.5	Calcium ion binding	Bone mineralization
16	P04004	Vitronectin	0.61	0.66	—	Collagen binding	Cell adhesion
17	P02647	Apolipoprotein A1	0.25	0.53	—	Amyloid-beta binding	Adrenal gland development
18	P02787	Serotransferrin	0.38	—	—	Ferric iron binding	Iron ion homeostasis
19	P01024	Complement C3	0.43	—	—	C5L2 anaphylatoxin chemotactic receptor binding	amyloid-beta clearance
20	P02774	Vitamin D-binding protein	0.38	—	—	Actin binding	Vitamin D metabolic process
21	P02743	Serum amyloid p component	0.46	—	—	Calcium ion binding	Acute-phase response
22	P06396	Gelsolin		—	0.56	Actin binding	Actin filament capping
23	P02768	Serum albumin	0.26	—	0.14	Antioxidant activity	Bile acid and bile salt transport

#### Common Proteins Identified and Validated in MM BMIF and Serum Samples

Multipronged quantitative proteomic approaches yielded a total of 279 and 116 non-redundant differentially abundant proteins in BMIF and serum samples of MM study cohorts, respectively. A total of 55 proteins with statistically significant differential abundance were identified to be common in both MM BMIF and serum study cohorts. Interestingly, 41 proteins showed a similar pattern of differential expression and among these, 23 proteins were up-regulated and 18 proteins were down-regulated.

### Bioinformatic Analysis

By employing online web applications such as PANTHER, DAVID and IPA, we tried to extract the biological information from the differentially regulated proteins identified in MM BMIF. A total of 279 statistically significant and dysregulated non-redundant proteins identified from multipronged proteomic analyses were investigated. The key biological processes found to be altered in MM were complement activation, receptor-mediated endocytosis, platelet degranulation, proteolysis, immune response, blood coagulation, positive regulation of B-cell activation, endopeptidase activity, phagocytosis recognition, acute phase response, cholesterol efflux, innate immune response (**Figure 6B**). Various cancer-related pathways emerged to be altered in MM such as integrin signaling pathway, apoptosis signaling pathway, P-53 pathway, Wnt signaling pathway and FAS signaling pathway (**Figure 6C**). Different molecular functions, biological processes, cellular components and proteins classes were found to be involved in MM pathogenesis (**Figure S1**). Likewise, many canonical pathways, upstream regulators and toxicology functions were identified, through IPA software, to be associated with MM BMIF samples (**Figure S2**). IPA analysis identified the connection of several upstream regulators like HNF4A, dexamethasone, lipopolysaccharide, PPARA, TGFB1 *etc*., for the differentially regulated proteins of MM BMIF.

Similarly, differentially expressed proteins in MM serum samples were also analyzed along the same lines as described above. A total of 116 statistically significant dysregulated non-redundant proteins identified from multipronged quantitative proteomic approaches were subjected to bioinformatic analysis. The majority of the key biological processes that were altered in BMIF were also found to be dysregulated in serum. These processes include platelet degranulation, endopeptidase activity, receptor-mediated endocytosis, acute phase response, complement activation, innate immune response, B cell receptor signaling and phagocytosis (**Figure 7B**). Moreover, various cancer-related pathways emerged to be altered in MM such as angiogenesis, integrin signaling pathway, Wnt signaling pathway and EGF receptor signaling pathway (**Figure 7C**). Furthermore, different molecular functions, biological processes, cellular components and protein classes were also found to be involved in MM pathogenesis (**Figure S3**). In addition, IPA based analysis revealed many canonical pathways, upstream regulators and toxicology functions to be associated with MM serum samples (**Figure S4**). IPA analysis identified the involvement of several upstream regulators like IL6, nitrofurantoin, HNF1A, HNF4A, *etc*., for the differentially regulated proteins of MM serum.

### Verification Experiments

To verify the differentially expressed proteins found in the MM BMIF discovery phase of our study, we performed the verification experiments using two approaches *viz*. the western blotting and the mass spectrometry based SRM assay. Western blotting was performed for Kininogen 1, alpha-1-antitrypsin, vitronectin, gelsolin, apolipoprotein A1, and transferrin. The immunoblot data indicated a consistent pattern of differential expression as observed in discovery phase data (**Figure S5**). We also performed the SRM verification for some of the proteins that had not been verified through the western blotting due to the unavailability of antibodies in our lab and to adopt this cost-effective SRM verification strategy. Proteins such as ceruloplasmin, haptoglobin, apolipoprotein A-IV, alpha-1-acid glycoprotein 1, beta-2-glycoprotein, vitronectin, plasminogen, serum amyloid p-component, complement C3, transferrin, apolipoproteinA1, and fibrinogen alpha chain were verified by this approach (**Figure S6**). The results obtained in the SRM based verification were in good agreement with the discovery phase data.

Similarly, for selected proteins that are differentially expressed in MM serum, immunoblotting was performed for their verification. These proteins include haptoglobin, kininogen 1, zinc alpha-2-glycoprotein, gelsolin, apolipoprotein A1, and transferrin (**Figure S7**). Moreover, the proteins which are mentioned above along with few other proteins such as alpha-1-antitrypsin, ceruloplasmin, plasminogen, serum amyloid A1, complement C3, vitamin D binding protein, serum amyloid P component and alpha-1-antichymotrypsin were also verified by SRM based approach **(** **Figure S8**). The findings of immunoblot and SRM based assays were consistent with our discovery phase observations concerning either up-regulation or down-regulation of the above mentioned proteins.

### Validation Experiments

**Figure 8:**
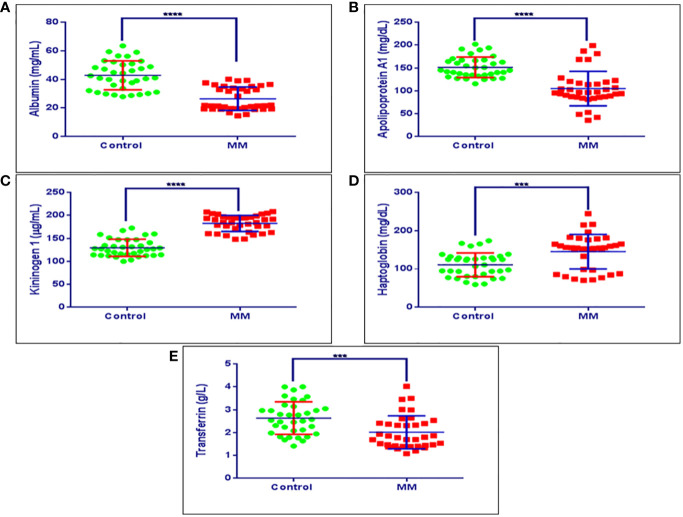
Validation experiments for some of the candidate proteins using ELISA. **(A)** Concentration ranges of serum albumin in healthy controls (42.80 mg/ml ± 10.13 mg/ml), MM patients (26.45 mg/ml ± 8.14 mg/ml). **(B)** Concentration ranges of serum apolipoprotein a1 in healthy controls (152.05 mg/dl ± 30.61 mg/dl), MM patients (105.97 mg/dl ± 38.80 mg/dl). **(C)** Concentration ranges of serum kininogen 1 in healthy controls (139.38 µg/dl ± 28.98 µg/dl), MM patients (173.95 µg/dl ± 26.60 µg/dl), **(D)** Concentration ranges of serum haptoglobin in healthy controls (110.86 mg/dl ± 30.99 mg/dl), MM patients (145.28 mg/dl ± 45.03 mg/dl). **(E)** Concentration ranges of serum transferrin in healthy controls (2.64 g/L ± 0.69 g/L), MM patients (1.76 g/L ± 0.48 g/L). Healthy control serum samples (n = 36), MM patients serum samples (n = 36). ****p-value ≤ 0.0001, ***p-value ≤ 0.001.

The BMIF, as well as serum proteins that showed statistically significant differential abundance in the discovery phase and verified for their expression by immunoblotting or SRM based approach were further validated in an external cohort of MM patients’ serum samples by ELISA (MM = 36, Control = 36). ELISA based validation experiments were performed for proteins *viz*., haptoglobin, kininogen 1, apolipoprotein A1, transferrin and albumin. Among these proteins, haptoglobin and kininogen 1 were found to be up-regulated, whereas apolipoprotein A1, transferrin and albumin proteins were down-regulated in MM samples. Serum albumin in MM patients was found to be at 26.45 mg/ml ± 8.14 mg/ml, while in healthy controls it was at 42.80 mg/ml ± 10.13 mg/ml. Serum apolipoprotein A1 in MM patients was at a concentration of 105.97 mg/dl ± 38.80 mg/dl whereas in healthy controls it was at 152.05 mg/dl ± 30.61 mg/dl concentration. The concentration range of serum kininogen 1 in MM patients was 173.95 µg/dl ± 26.60 µg/dl whereas in healthy controls it was 139.38 µg/dl ± 28.98 µg/dl. MM patients had a concentration of 145.28 mg/dl ± 45.03 mg/dl for serum haptoglobin, which was found to be decreased in healthy controls at 110.86 mg/dL ± 30.99 mg/dL. Further, the concentration of serum transferrin in MM patients was at 1.76 g/L ± 0.48 g/L whereas in healthy controls it was found to be 2.64 g/L± 0.69 g/L. The concentration range of these proteins in healthy controls as well as in MM samples is showed in **Figure 8**. Further, these proteins were investigated for their ability to discriminate the MM patients from healthy controls by building the Receiver Operating Characteristic (ROC) curve analysis (**Figure S9**).

## Discussion

MM is the second most common hematological malignancy associated with plasma cell deformation and ultimately leads to bone marrow resorption. Currently, MM diagnosis primarily depends on the protein markers like serum albumin and B2M which are lesser specific at the early stages of this disease. Therefore, finding the novel and sensitive diagnostic and therapeutic markers for MM has a tremendous potential to improve patient survival. BMIF is the proximal biofluid for hematological malignancies and could serve as a potential source for identifying diagnostic, prognostic and therapeutic markers for MM. But unfortunately, bone marrow aspiration is an invasive and tedious procedure that puts patients through considerable anxiety and stress. Hence, to overcome this problem, for the first time, we identified and verified a set of differentially abundant proteins in MM BMIF and serum samples using multipronged quantitative proteomic approaches. Further, we selected a set of common proteins that showed a similar pattern of differential abundance in both MM BMIF as well as serum. Finally, we validated a panel of potential candidate biomarkers in a large cohort of serum samples that is associated with MM.

The bioinformatics analysis of the significant differentially abundant proteins found to be involved in many biological processes, some of which are elaborated henceforth in context to the proteins identified in this study. Platelet degranulation has been reported as a major biological process being altered in various cancers due to differentially expressed proteins (37, 38). In this study, platelet degranulation proteins such as complement C3, serum albumin, alpha-2-HS-glycoprotein, transferrin, extracellular matrix protein 1 and tetranectin were seen to be down-regulated in both MM BMIF as well as serum. A report from Gay et al. infers that platelet activation followed by degranulation serves as an important phenomenon in cancer (37). They have suggested that platelets in the circulatory system help the cancer cells to evade the immune response and enhance their malignant potential. We have found some proteins involved in this process to be down-regulated which is in agreement with the earlier report by Gay and co-authors (37). Albumin is one of the well-established biomarkers and is used in the international staging for the MM, the lower levels of albumin is associated with greater disease severity (39, 40). It regulates the platelet degranulation *via* its down-regulation, which is also evident from a similar expression pattern found in our study.

MM is a plasma cell cancer and these cells are derived from B cells. Hence, any alterations in the B-cell pathways such as the positive regulation of B cell activation and B cell receptor (BCR) signaling pathway, as observed in this study, could play an important role in MM development and progression. Interestingly, differentially expressed proteins *viz*., Ig kappa chain C region, Ig mu chain C region, Ig alpha-1 chain C region and Ig heavy chain V-III region involved in these pathways depicted an up-regulated expression pattern in both biological fluids. BCR signaling plays a key role in the maintenance and development of B cells. The pathways altered downstream of BCR signaling would lead to the proliferation and survival of the B cells. The differentially expressed proteins associated with the BCR signaling pathway found in our study were positively modulated, which could lead to proliferative signals leading to the development of MM as explained by Choi & Kipps (41). Various lymphomas utilize BCR signaling as a key oncogenic pathway to promote the proliferation and survival of B cells (42–44).

Complement activation is an important cause for inflammation and a series of experiments have proved that it could lead to tumor progression (45). Complement component C3-deficient mice were found to escape chemically induced carcinogenesis in different tissues due to reduced inflammation (46). They also identified the long pentraxin (a humoral component of innate immunity) which acts as a negative regulator of complement activation and cancer-related inflammation and further showed that PTX3 knockout mice were prone to carcinogenesis. In our study, we observed that the majority of proteins related to complement activation pathways such as Ig kappa chain C region, Ig J chain, Complement C4-B, Ig gamma-1 chain C region and Protein IGKV3-11 showed increased expression pattern.

Previous studies have reported that higher levels of intracellular cholesterol positively affect cancer cell proliferation and migration (47, 48). The positive regulation between the elevation of cellular cholesterol and tumorigenesis mechanism is not clearly reported and needs to be explored. Our study identified several proteins involved in cholesterol efflux such as apolipoprotein A-4, apolipoprotein A-1, apolipoprotein C-II, apolipoprotein M, apolipoprotein L1, apolipoprotein E, apolipoprotein D and apolipoprotein C1 to be down-regulated in MM BMIF and serum. Similarly, the lipoprotein metabolomic process was also altered and most of the proteins were the same as those found in cholesterol efflux.

Some of the acute phase proteins have been related to distinct cancers and also linked to their malignancy stages. Acute-phase proteins like alpha-1-acid glycoprotein 1, inter alpha- trypsin, serum amyloid A protein, alpha-1-antitrypsin, serum amyloid A-4 protein, C-reactive protein (CRP) showed up-regulation in this study. Previously, few reports demonstrated CRP levels to be elevated in squamous cell carcinoma of the esophagus and in adenocarcinoma and revealed that an increase in CRP levels correlated with tumor growth and metastasis (49–51). Ilhan et al. and Chan et al. demonstrated that CRP and serum amyeloid A-1 proteins were elevated in gastric cancer and SAA was also found to be elevated in recurrent gastric cancer patients (52, 53).

During the tumor progression, various innate immunity components are activated to minimize the inflammation caused by cancer (54, 55). These activated innate immunity components further positively affect the adaptive immune responses and help in targeting the cancer cells (54, 55). In our study, innate immunity-related proteins such as Ig J chain, Ig lambda-like polypeptide 5, Ig alpha-1 chain C region, serum amyloid A-1 protein, Ig kappa chain C region, beta-2-microglobulin and complement C4-B showed up-regulation. The B2M protein level is elevated in MM and is an approved diagnostic marker for MM detection that was found to be up-regulated in our MM serum samples as well. Various other biological processes such as endopeptidase activity, receptor-mediated endocytosis, blood coagulation, phagocytosis and immune response were also found to be altered due to the differentially expressed proteins suggesting their potential role in the MM.

Cancer-associated pathways such as integrin signaling pathway, inflammation mediated by chemokine and cytokine signaling pathway, Wnt signaling pathway and FAS signaling pathway appeared to be altered in both MM BMIF as well as serum. The integrin signaling pathway plays an important role in cancer growth, metastasis, and therapy resistance in the tumor cells as well as in stromal cells. Integrins involve in the interaction of cells with their local environment and translate the external chemical response into a concerted intracellular response (56). Chemokine signaling pathways regulate the immune responses, angiogenesis, epithelial cell growth and survival. Chemokines are also critical for cancer progression and play an important role in the tumor microenvironment (57). Likewise, cytokine inflammation signaling pathways linked to chronic diseases such as obesity, heart diseases and cancer  (58). Similarly, various research studies reveal that the Fas/FasL signaling and Wnt signaling pathway plays a crucial role in the impairment of cancer cells and leads to apoptosis resistance and tumor progression (59, 60). Hence, alterations of these pathways due to the dysreulated proteins could have an important role in the MM cancer progression.

Further, we performed the verification experiments using two approaches *viz*., the western blotting and the mass spectrometry based SRM assay to verify the proteins differentially regulated in the discovery phase. A panel of 15 Proteins *viz*., kininogen 1, alpha-1-antitrypsin, vitronectin, gelsolin, transferrin, ceruloplasmin, haptoglobin, apolipoprotein A-IV, alpha-1-acid glycoprotein 1, beta-2-glycoprotein, plasminogen, serum amyloid p-component, complement C3, apolipoprotein A1, and fibrinogen alpha chain were verified in MM BMIF. Similarly, a panel of 14 proteins *viz*. haptoglobin, kininogen 1, alpha-1-antitrypsin, zinc-2-alpha glycoprotein, gelsolin, apolipoprotein A1, transferrin, ceruloplasmin, plasminogen, serum amyloid A1, complement C3, vitamin D binding protein, serum amyloid P component and alpha-1-antichymotrypsin were verified in an external cohort of serum samples. Finally, we validated a panel of five of the verified proteins such as haptoglobin, kininogen 1, transferrin and apolipoprotein A1 along with known MM biomarker, albumin in a large cohort of serum samples using ELISA. This panel of proteins could be useful in the diagnosis and understanding of the pathophysiology of MM. The relevance of each protein in the potential biosignature panel of MM identified in this study is discussed henceforth.

Haptoglobin is an acute phase alpha glycoprotein comprised of two alpha and two beta subunits and tasked with hemoglobin clearance upon erythrocyte lysis. Haptoglobin is also known to carry out several other functions including antioxidant, anti-inflammatory and immune response regulation (61). Moreover, a recent study has shown that elevated levels of serum haptoglobin regarded as a diagnostic and prognostic marker of non-small-cell lung carcinoma (NSCLC). The study indicated that higher levels of haptoglobin have positively correlated with disease progression as well as distant organ metastasis in NSCLC (62). Furthermore, in another study, haptoglobin alpha subunit was found to be highly overexpressed in serum samples of ovarian cancer patients as compared to controls. The authors of the study projected haptoglobin alpha subunit as a complementary diagnostic marker along with established CA 125 protein (63). Similarly, in our study, we also observed consistent up-regulation of haptoglobin in MM BMIF and serum samples as compared to controls. Furthermore, the up-regulation of haptoglobin was observed in all three approaches *viz*., 2D-DIGE, iTRAQ, SWATH in MM BMIF and serum samples suggesting a potential candidate for MM. Overall, the MM serum and BMIF haptoglobin expression profiles were in good agreement with previous reports.

Alternate splicing of kininogen 1 transcript produces high molecular weight kininogen (HMWK) and low molecular weight kininogen (LMWK). The HMWK is involved in blood coagulation as well as the proteolytic release of bradykinin peptide (64). Bradykinin plays an important role in several physiological activities such as antimicrobial, smooth muscle contraction and release of inflammatory mediators like prostaglandins (65). Moreover, bradykinin is also known to interact with EGFR and stimulate downstream signaling pathways to enhance cell invasion as well as promote angiogenesis through elevated VEGF expression in glioma (66, 67). Furthermore, higher expression of kininogen 1 is recently reported as a serum biomarker of advanced colorectal adenoma, colorectal cancer and prognostic marker of oral cancer (68, 69). Interestingly, in our study, we also noticed elevated levels of kininogen 1 in BMIF as well as serum samples of MM patients as compared to the controls in discovery as well as validation cohorts. The findings were consistent with earlier reports of higher expression of kininogen 1 under the malignant condition and could be projected as a potential candidate biomarker for MM.

Transferrin is an iron transport protein present in blood serum. The total concentration of blood transferrin usually shown as a total iron-binding capacity of serum (TIBC). Lower TIBC leads to hemolytic anemia in which red blood cells destroyed very early stages (70). Interestingly, it is well known that anemia is a common characteristic feature of MM (71). Furthermore, it is also reported that low TIBC was significantly associated with lower disease-free survival as well as the overall survival rate of gastric cancer patients (72). Likewise, Hodgkin’s lymphoma is also reported to be associated with a low serum iron concentration and reduced transferrin saturation (73). In contrast to the above mentioned studies, few studies have also shown that transferrin could be a positive regulator for the cancer cells proliferation by acting as an autocrine regulator (74). In our study, we observed reduced levels of transferrin in MM BMIF and serum samples as compared to controls. Since, transferrin is associated with anemia which is a well known characteristic feature of MM, it could be a potential protein marker for MM.

Apolipoprotein A-1 is a multifunctional component of high-density lipoprotein (HDL) involved in inflammation and immune response regulation apart from cholesterol trafficking. Recently, many studies have observed that apolipoprotein A-1 levels are altered upon cancer development and indicated that it could serve as a useful marker for diagnosis, prognosis as well as risk stratification of cancer patients (75). It’s been also reported that reduced levels of apolipoprotein A-1 have been observed in several cancers including adenocarcinoma of the gastrointestinal tract, lung and breast adenocarcinoma, early-stage ovarian cancer, cervical cancer and lymphoblastic leukemia (76–82). Furthermore, it’s also documented that apolipoprotein A-1 levels were significantly lowered upon the metastatic recurrence of the liver, breast, endometrial and cervical cancer (83–86). Moreover, a prominent association of apolipoprotein A-1 was also revealed in the prognosis of several cancers (87). In our study, we also noticed reduced levels of apolipoprotein A-1 in MM BMIF and serum samples as compared to controls. The findings were consistent with earlier reports but, further studies are needed to probe the precise mechanism of apolipoprotein A-1 role in MM manifestation.

Albumin is a globular protein synthesized in the liver and performs the primary function of regulating the oncotic pressure of blood apart from acting as a carrier protein for various hormones, vitamins, and enzymes. Albumin constitutes over 60% of total blood plasma protein and its differential abundance levels have been intrinsically associated with several ailments. Importantly, in one of the study, hypoalbuminemia, a condition of reduced albumin levels have been detected as a marker of the advanced stage as well as higher cancer burden in MM patients (88). Authors have observed that the albumin levels ≤29.0 g/L indicate advanced disease stage of MM. Furthermore, in another study, reduced levels of serum albumin (<3.5 g/dl) projected as a significant prognosis factor in symptomatic MM (39). In our study, we also detected lower levels of albumin in MM BMIF as well as serum samples as compared to the controls in all three proteomic approaches. The findings of our study support the already confirmed fact that decreased abundance levels of albumin is associated with MM disease manifestation and hence, could be employed as an important member of the panel of proteins identified as a diagnostic marker in our study that could be translated to future clinical settings with higher confidence.

## Conclusions

MM is a disease that is primarily diagnosed in the advanced stages where the medical interventions for the treatment are very limited. Early diagnosis of MM can be crucial as the chances of better disease management by the clinicians can increase the overall survival expectancy of the MM patients. Protein-based makers of MM can comply with finding the biosignature which could be developed as early predictor of this disease. Our study design was to identify a biosignature panel of proteins from the BMIF and serum samples of MM patients and cross-verified its abundance in an independent cohort of serum samples of MM patients as the serum is an easily obtained diagnostic biofluid. Using multipronged proteomic approaches, we identified 279 and 116 non-redundant differentially expressed proteins in MM BMIF and serum respectively. A verification phase of experiments in an external cohort of BMIF and serum samples confirmed a set of 15 and 14 proteins respectively. Finally, a combined panel of four common proteins namely haptoglobin, kininogen 1, transferrin and apolipoprotein A1 along with albumin (an established biomarker for MM) were validated in a fresh cohort of serum samples and could be better and minimally invasive diagnostic, prognostic markers for MM. However, validation in a larger cohort of MM patients can further help to investigate the practical potentiality of these proteins as early diagnostic and prognostic markers. We believe that this panel of proteins could help in future MM disease management and thereby improving the survival expectancy of MM patients.

## Data Availability Statement

The datasets presented in this study can be found in online repositories. The names of the repository/repositories and accession number(s) can be found below: Proteome Xchange Consortium *via* the PRIDE partner repository (89) having identifier PXD008927.

## Ethics Statement

The studies involving human participants were reviewed and approved by Armed Forces Medical College, Pune and National Centre for Cell Science, Pune. The patients/participants provided their written informed consent to participate in this study.

## Author Contributions

Conceived the study: VC, TC, SR. Designed the study: VC, KT, TC, SS, VS, MS, SR. Performed the experiments: VC, FR. Compiled the data: VC, RT, KT, FR, DM, SR. Analyzed the proteomics data and performed the statistical and bioinformatics analysis: VC, RT, KT, FR, DM, SR. Drafted the manuscript: VC, RT, KT, TC, SS, FR, DM, MS, SR. Provided clinical samples: TC, SS, VS. Provided chemicals and reagents: SR. All authors contributed to the article and approved the submitted version.

## Funding

This research was supported by NCCS intramural funding and the Department of Biotechnology, Govt. of India, India (grant no. BT/PR10855/BRB/10/1330/2014).

## Conflict of Interest

Authors FR and DM are employed by Sciex, Gurgaon, India.

The remaining authors declare that the research was conducted in the absence of any commercial or financial relationships that could be construed as a potential conflict of interest.
