# Supplementary material for: Proteomic Alterations in Multiple Myeloma: A Comprehensive Study Using Bone Marrow Interstitial Fluid and Serum Samples
Source: Front Oncol. 2021 Jan 29;10:566804. doi: 10.3389/fonc.2020.566804 (PMC7879980; doi:10.3389/fonc.2020.566804)
Supplement: Supplementary file 1 [file Presentation_1.pptx]

## Slide 1
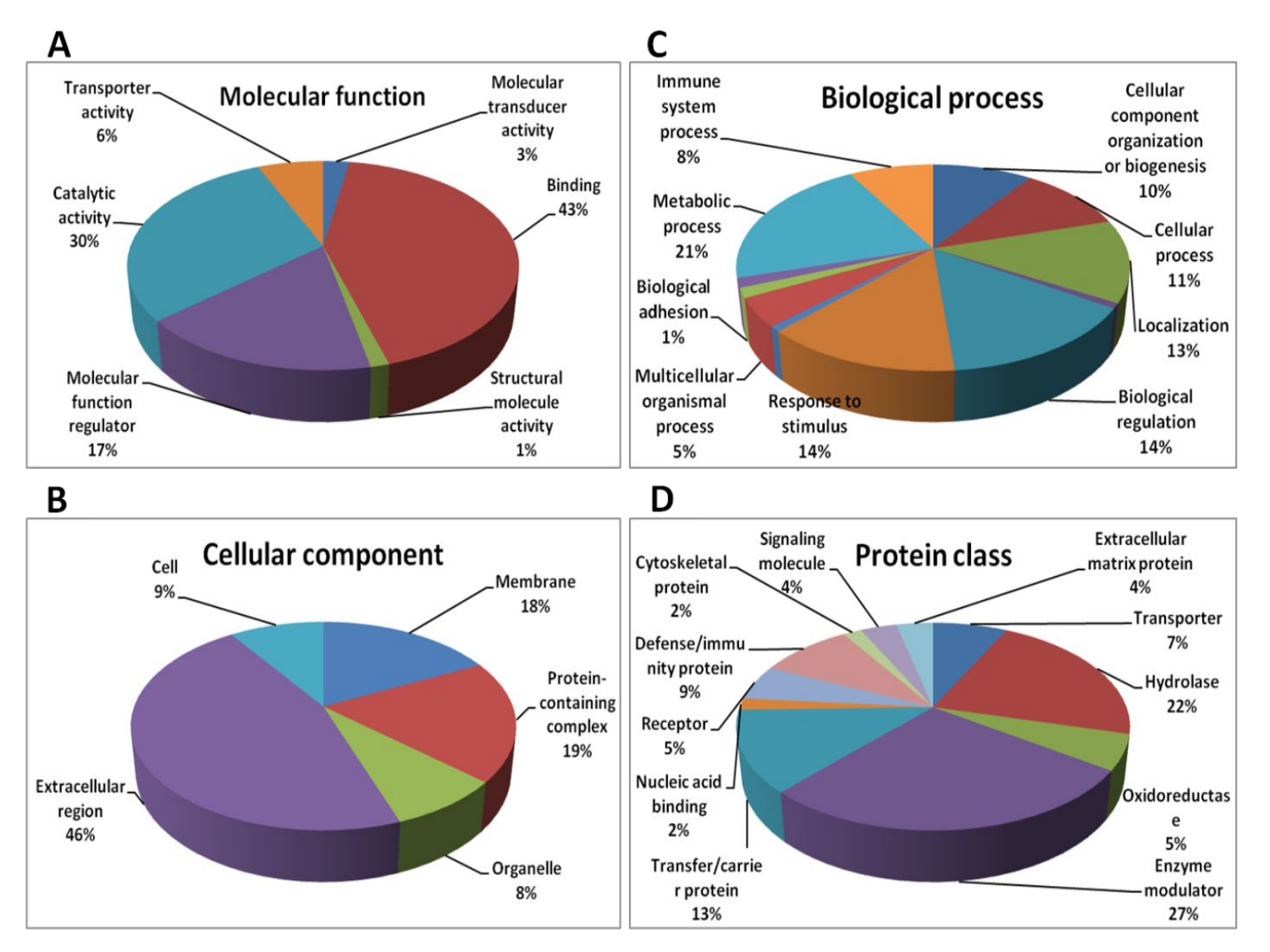

## Slide 2
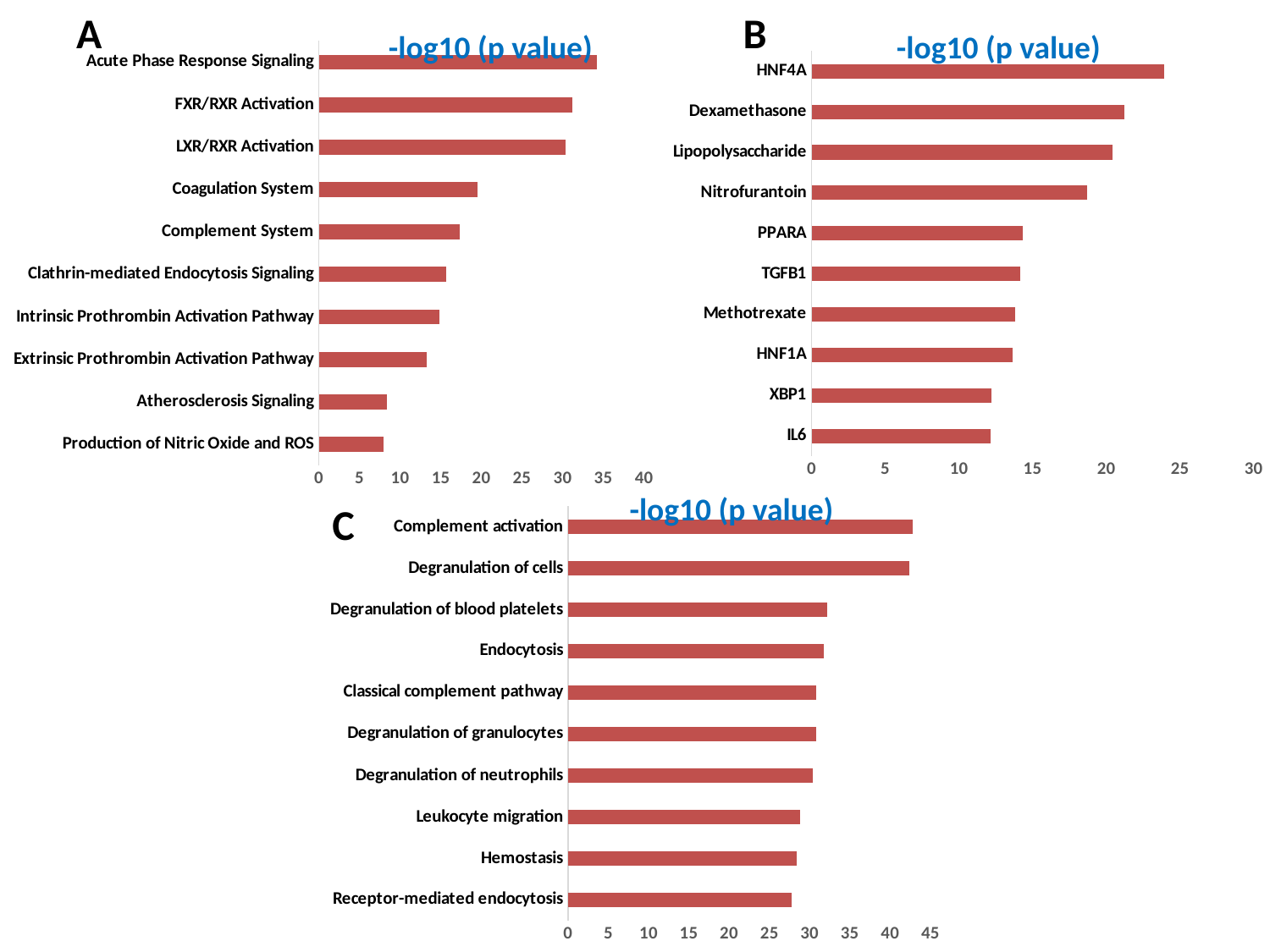

A
B
-log10 (p value)
-log10 (p value)
### Chart
| Category | |
|---|---|
| Production of Nitric Oxide and ROS | 7.98 |
| Atherosclerosis Signaling | 8.34 |
| Extrinsic Prothrombin Activation Pathway | 13.3 |
| Intrinsic Prothrombin Activation Pathway | 14.8 |
| Clathrin-mediated Endocytosis Signaling | 15.7 |
| Complement System | 17.3 |
| Coagulation System | 19.5 |
| LXR/RXR Activation | 30.3 |
| FXR/RXR Activation | 31.2 |
| Acute Phase Response Signaling | 34.2 |
### Chart
| Category | |
|---|---|
| IL6 | 12.171984935776024 |
| XBP1 | 12.1791420105603 |
| HNF1A | 13.636388020107855 |
| Methotrexate | 13.79317412396815 |
| TGFB1 | 14.177831920631982 |
| PPARA | 14.30539480106643 |
| Nitrofurantoin | 18.707743928643524 |
| Lipopolysaccharide | 20.431798275933005 |
| Dexamethasone | 21.221848749616356 |
| HNF4A | 23.89279003035213 |-log10 (p value)
C
### Chart
| Category | |
|---|---|
| Receptor-mediated endocytosis | 27.82390874094432 |
| Hemostasis | 28.373659632624957 |
| Leukocyte migration | 28.79048498545737 |
| Degranulation of neutrophils | 30.447331783887808 |
| Degranulation of granulocytes | 30.866461091629784 |
| Classical complement pathway | 30.872895201635192 |
| Endocytosis | 31.782516055786093 |
| Degranulation of blood platelets | 32.174573882232174 |
| Degranulation of cells | 42.40340290437354 |
| Complement activation | 42.76700388960785 |

## Slide 3
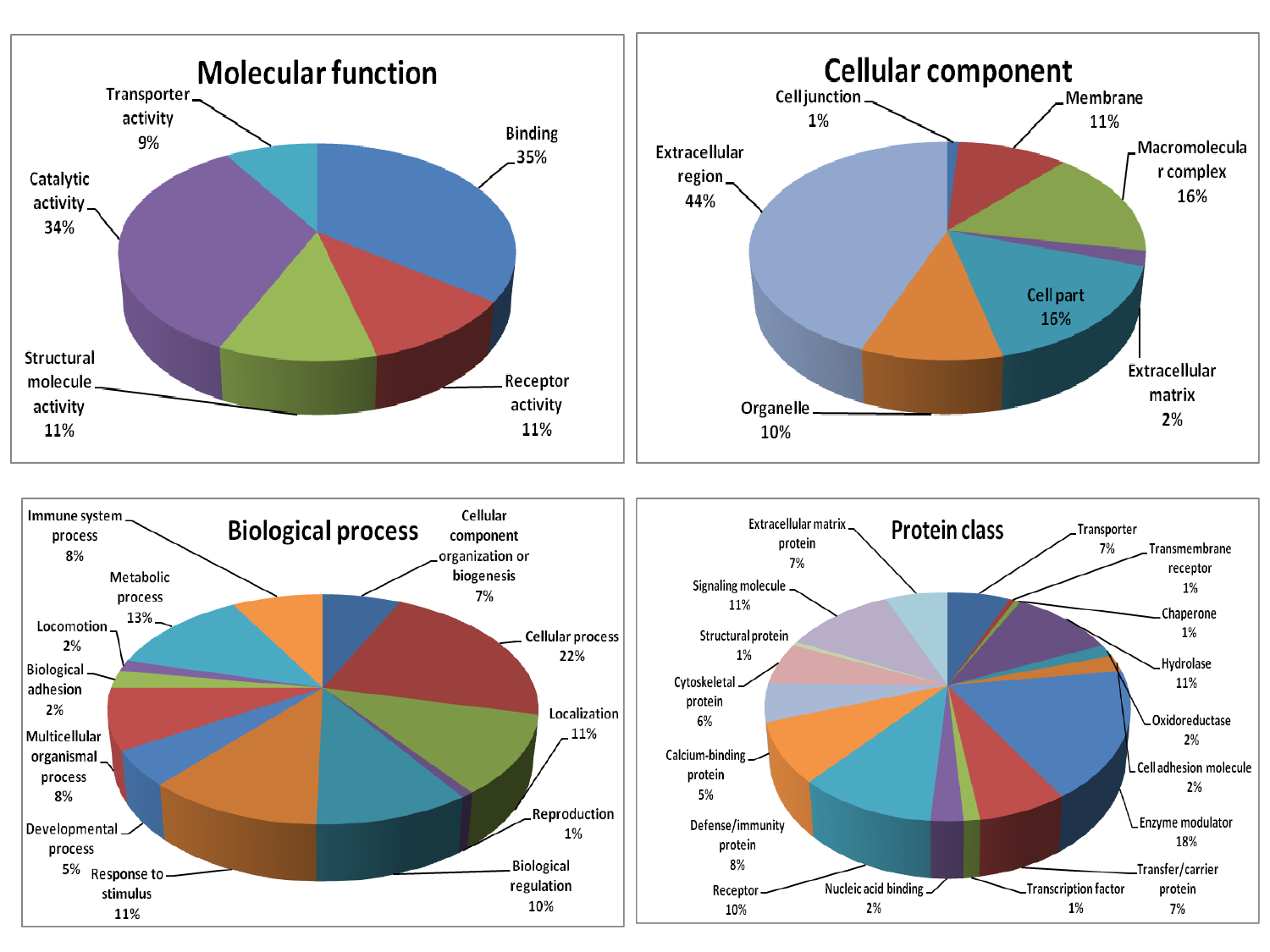

## Slide 4
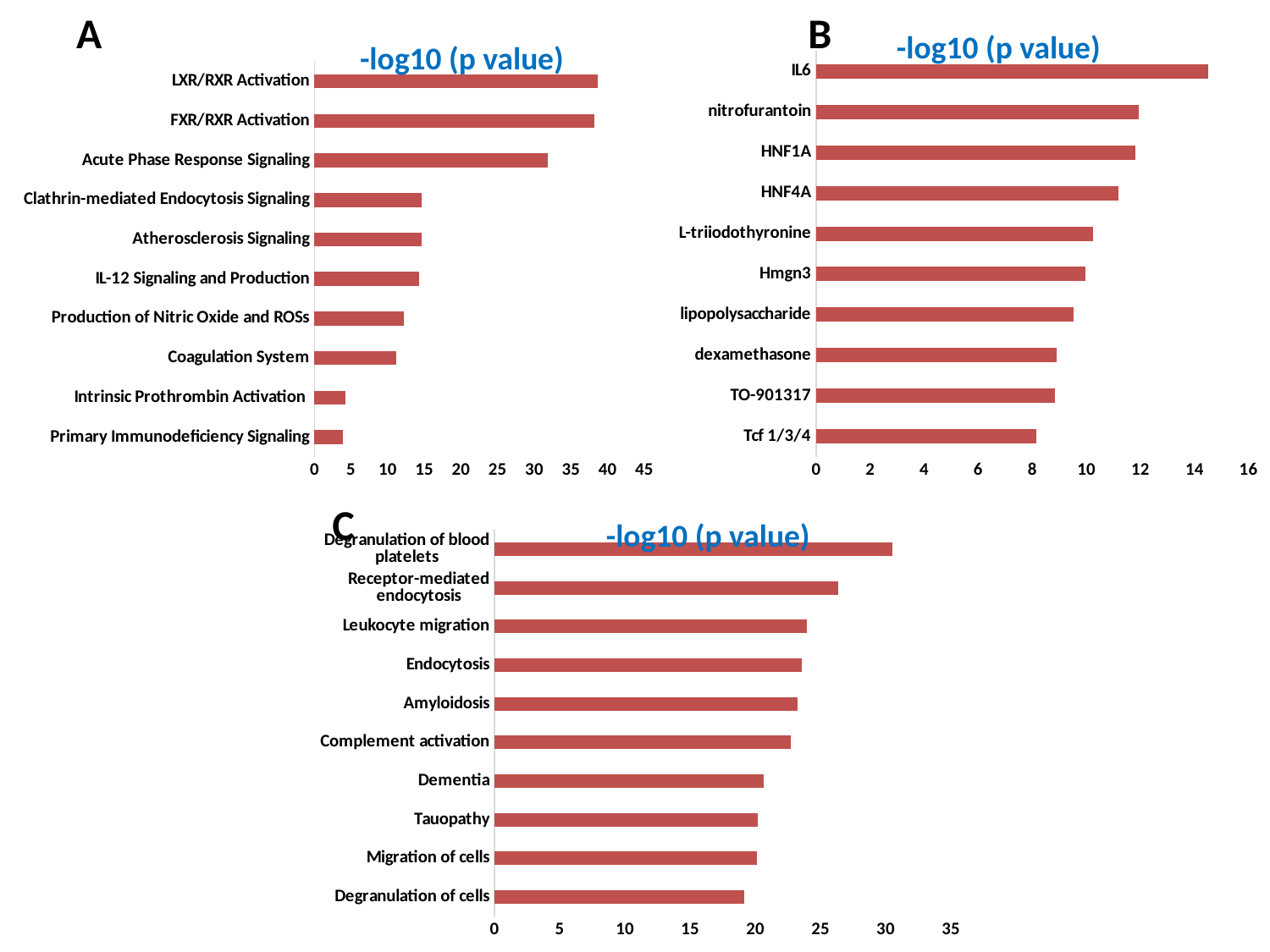

A
B
-log10 (p value)
-log10 (p value)
### Chart
| Category | |
|---|---|
| Tcf 1/3/4 | 8.149353764816933 |
| TO-901317 | 8.84163750790475 |
| dexamethasone | 8.910094888560602 |
| lipopolysaccharide | 9.512861624522813 |
| Hmgn3 | 9.978810700930062 |
| L-triiodothyronine | 10.233587152887601 |
| HNF4A | 11.195179321278838 |
| HNF1A | 11.8153085691824 |
| nitrofurantoin | 11.950781977329818 |
| IL6 | 14.515700160653214 |
### Chart
| Category | |
|---|---|
| Primary Immunodeficiency Signaling | 3.94 |
| Intrinsic Prothrombin Activation | 4.24 |
| Coagulation System | 11.2 |
| Production of Nitric Oxide and ROSs | 12.2 |
| IL-12 Signaling and Production | 14.3 |
| Atherosclerosis Signaling | 14.6 |
| Clathrin-mediated Endocytosis Signaling | 14.6 |
| Acute Phase Response Signaling | 31.9 |
| FXR/RXR Activation | 38.2 |
| LXR/RXR Activation | 38.7 |C
-log10 (p value)
### Chart
| Category | |
|---|---|
| Degranulation of cells | 19.170053304058364 |
| Migration of cells | 20.131355561605176 |
| Tauopathy | 20.193820026016112 |
| Dementia | 20.667561540084396 |
| Complement activation | 22.70996538863748 |
| Amyloidosis | 23.211124884224585 |
| Endocytosis | 23.546681659952963 |
| Leukocyte migration | 23.958607314841775 |
| Receptor-mediated endocytosis | 26.379863945026244 |
| Degranulation of blood platelets | 30.527243550682787 |

## Slide 5
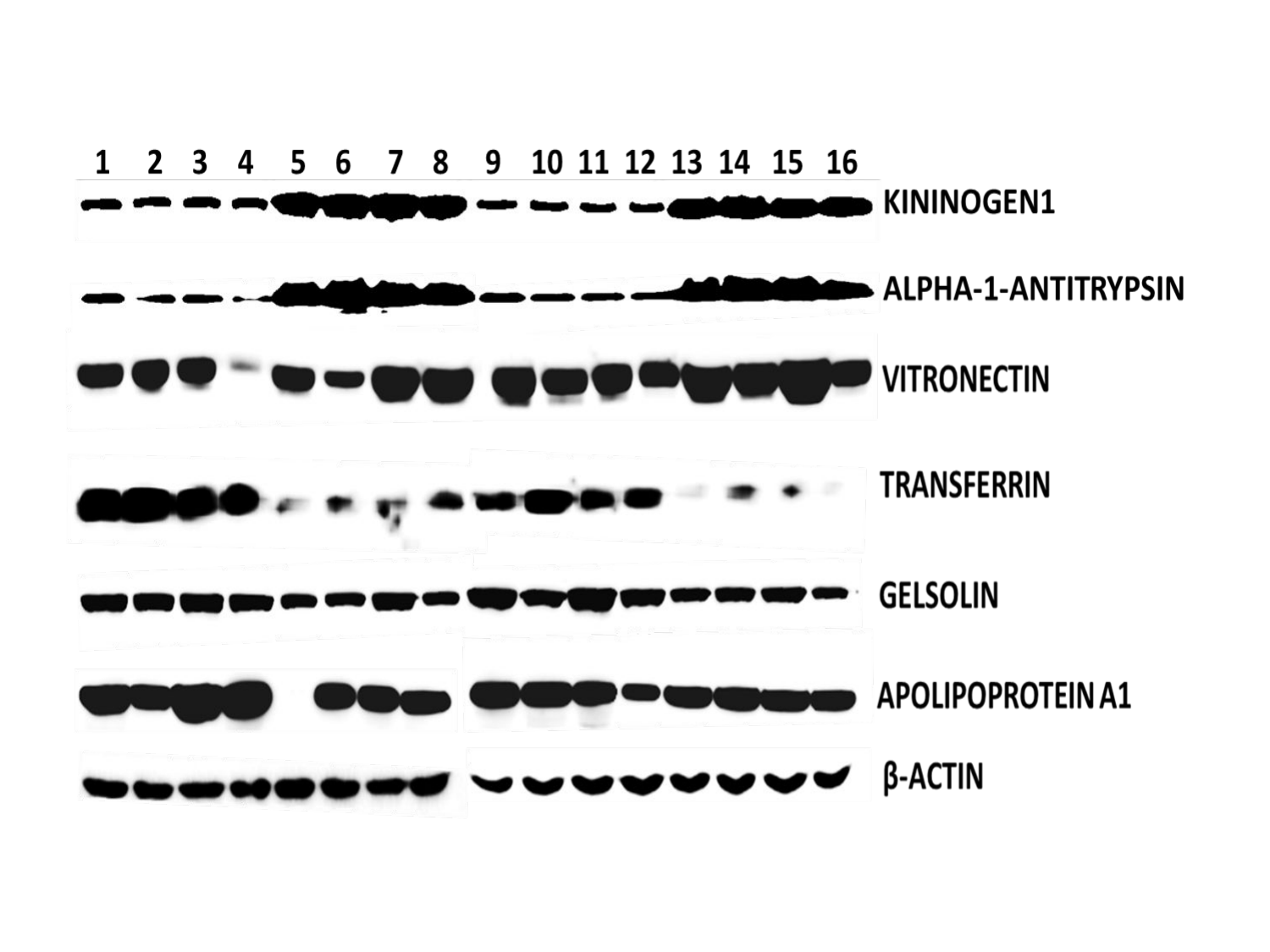

## Slide 6
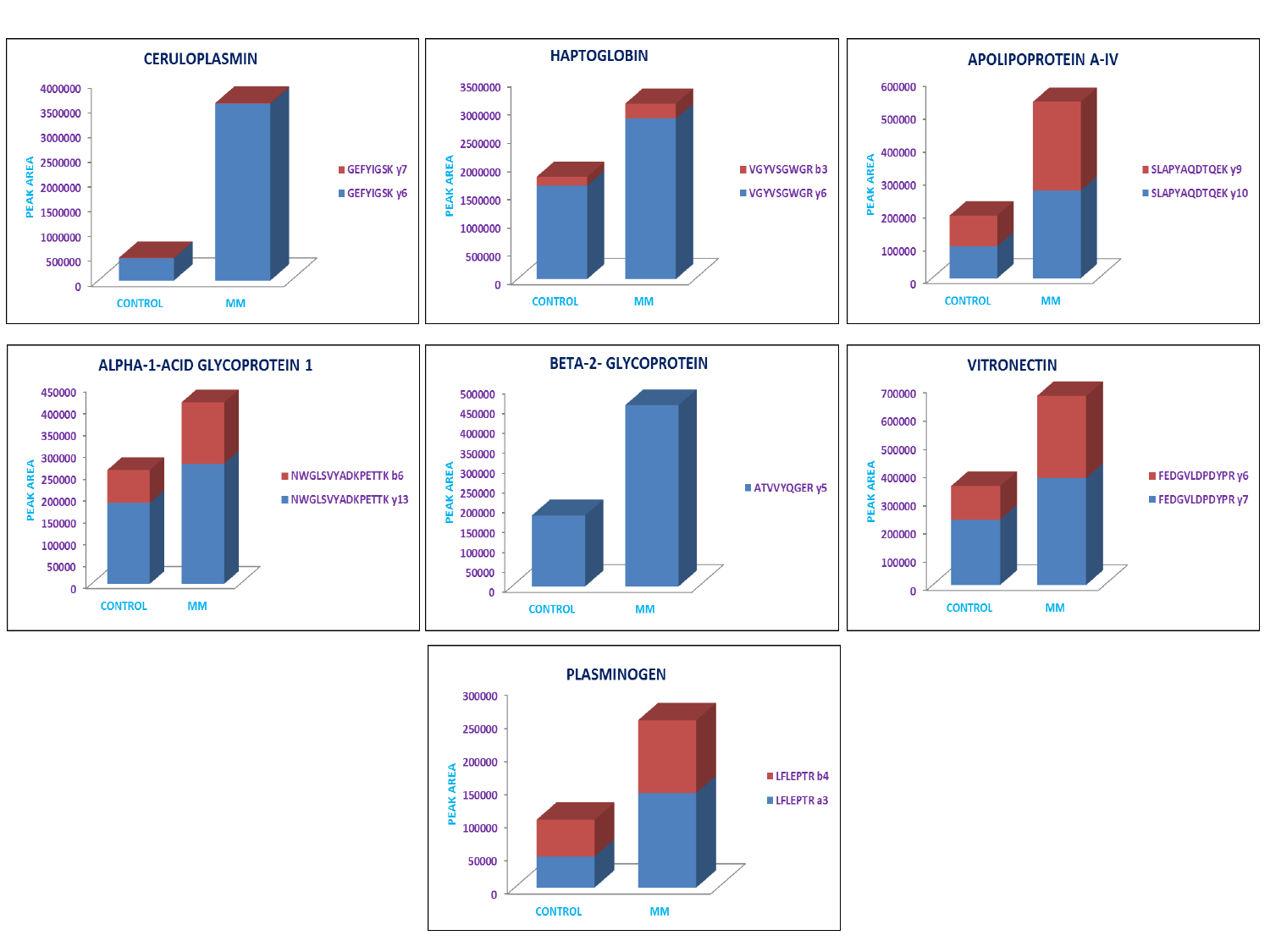

## Slide 7
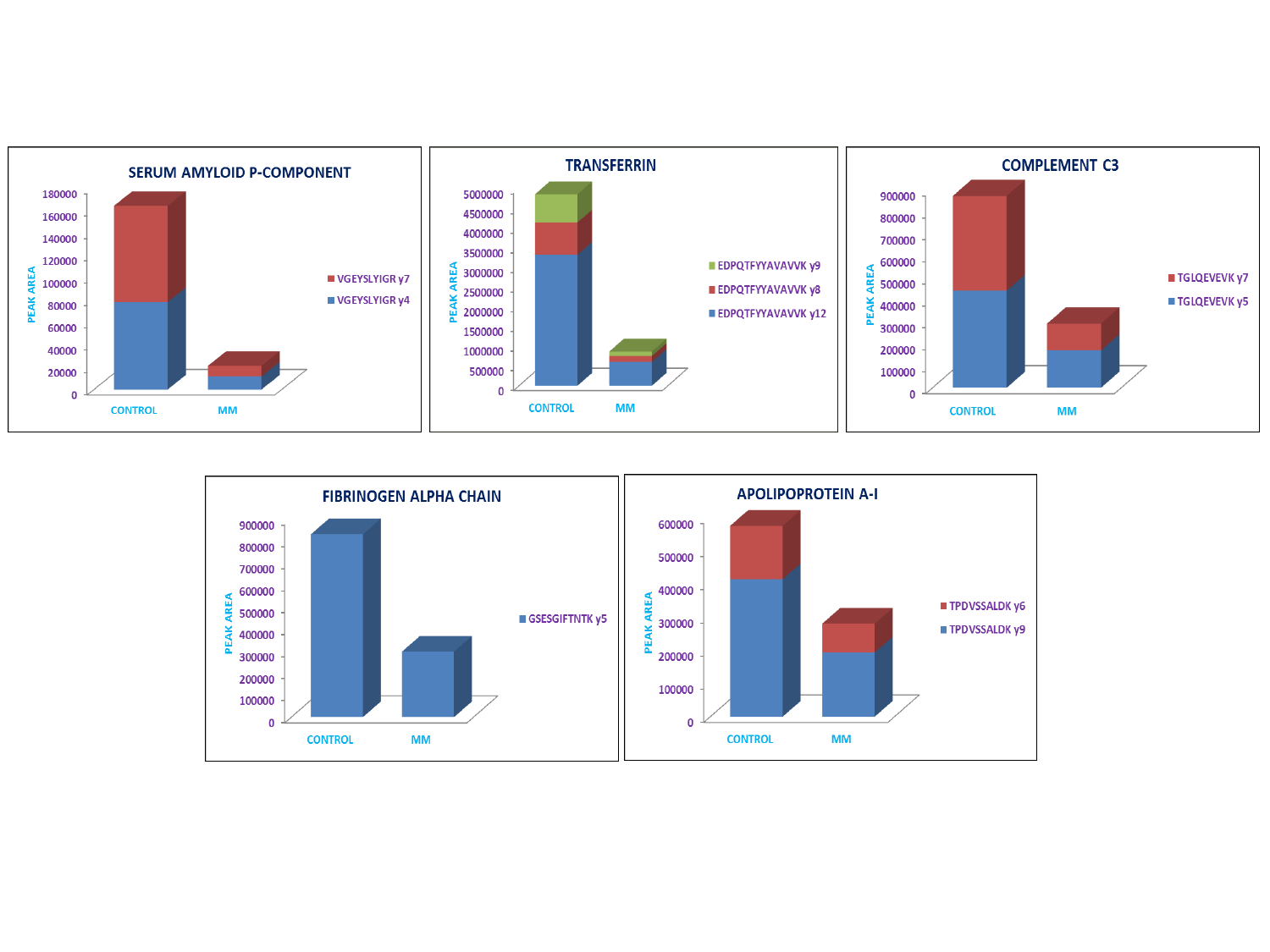

## Slide 8
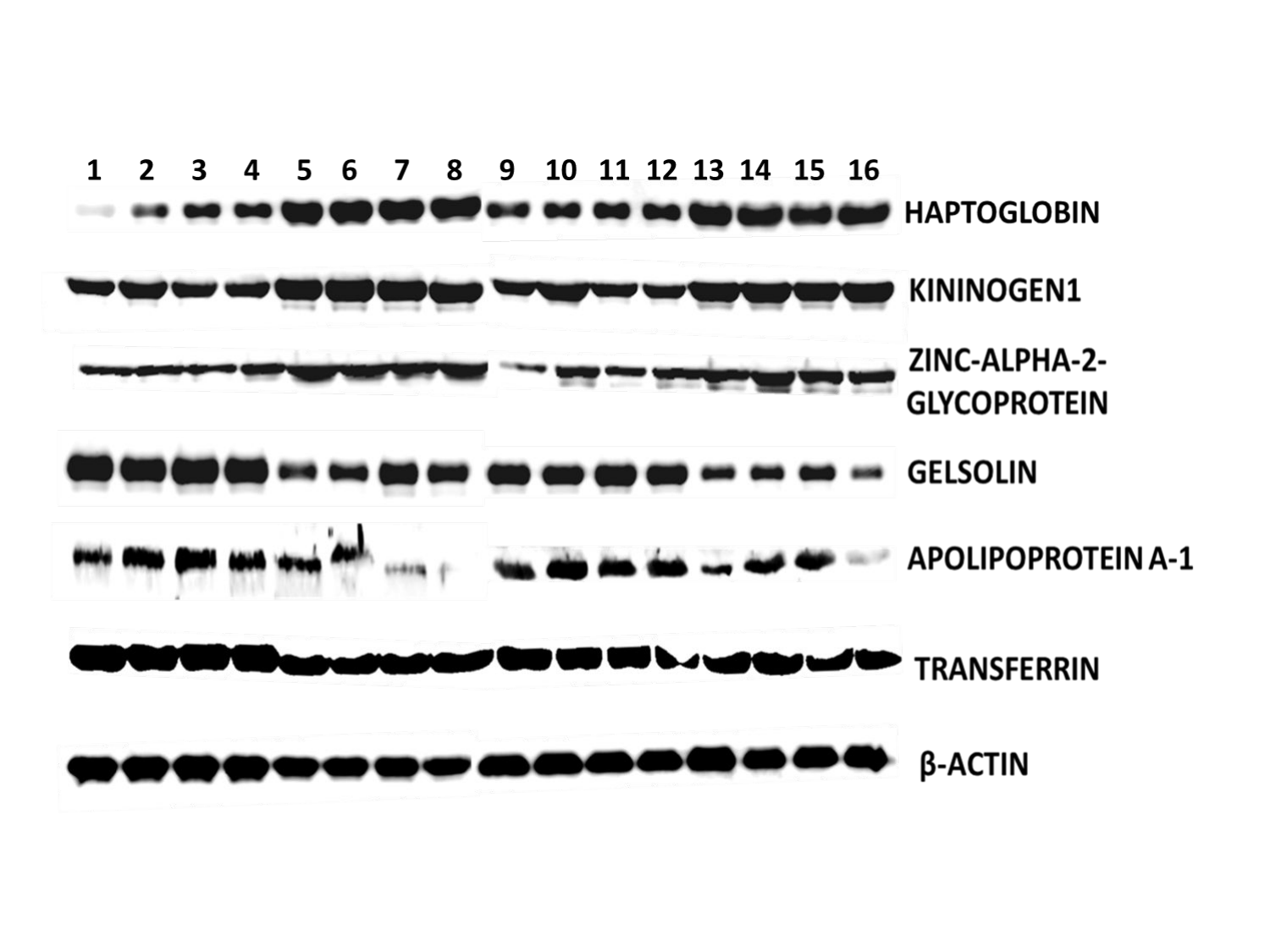

## Slide 9
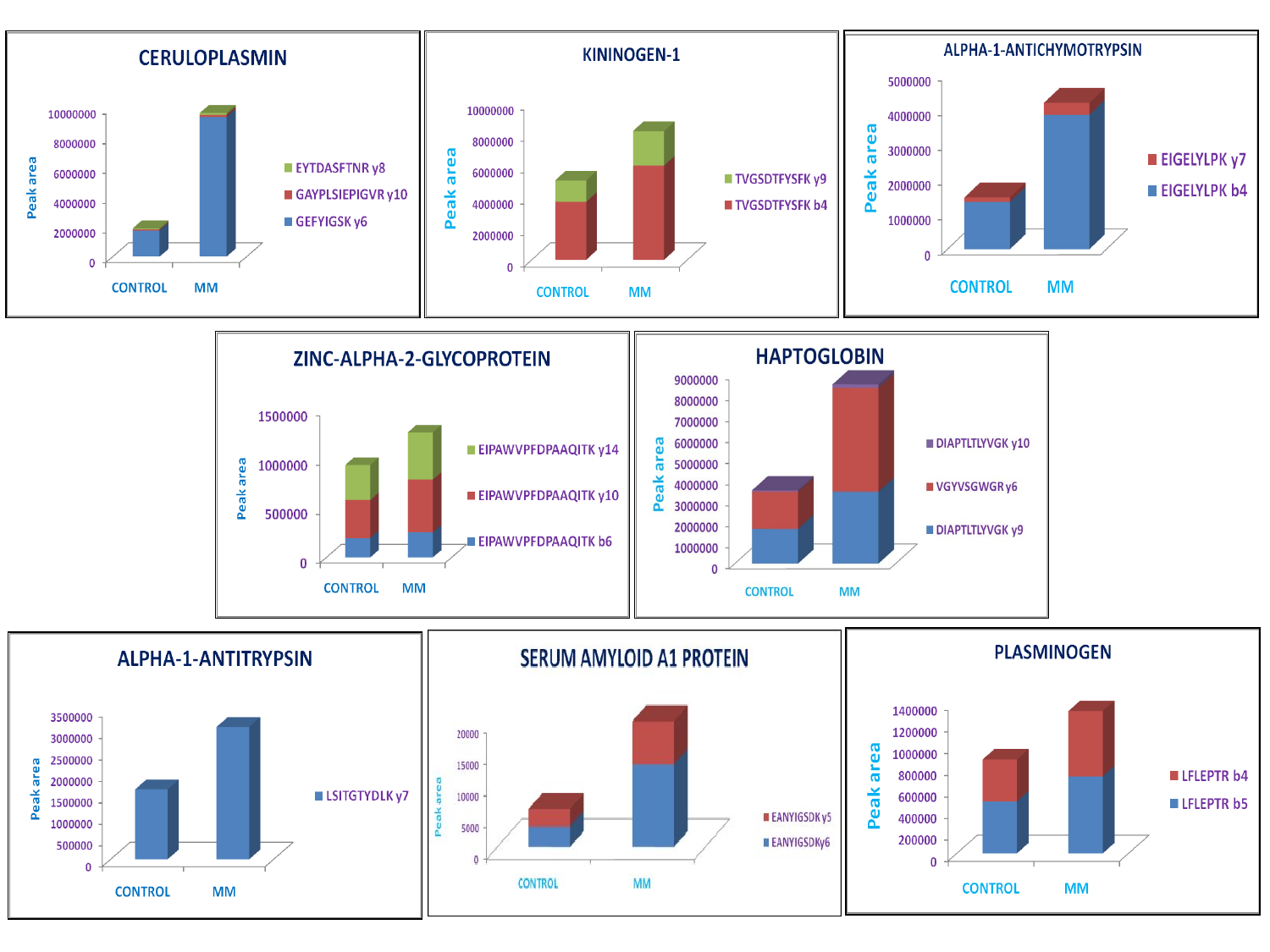

## Slide 10
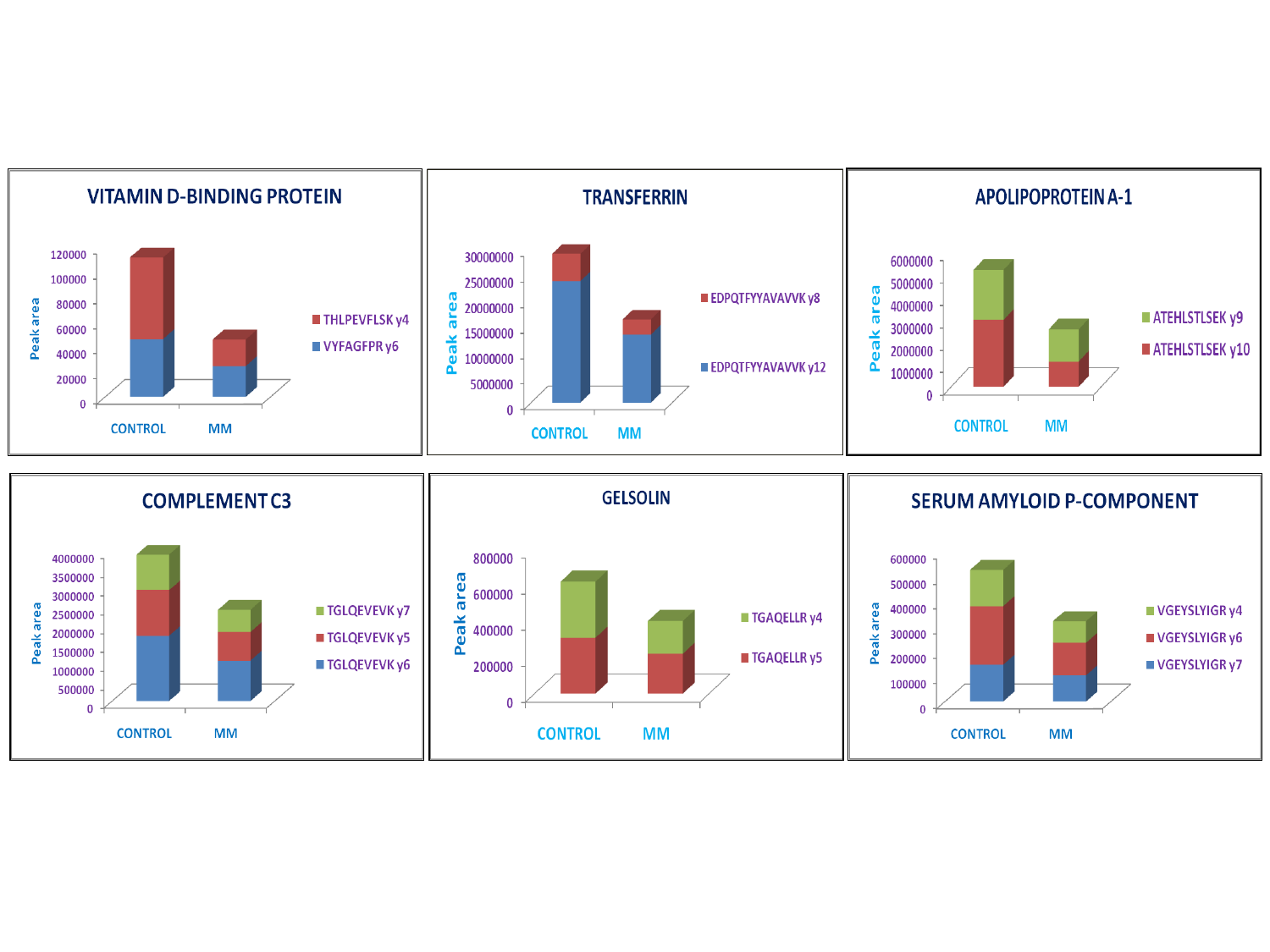

## Slide 11
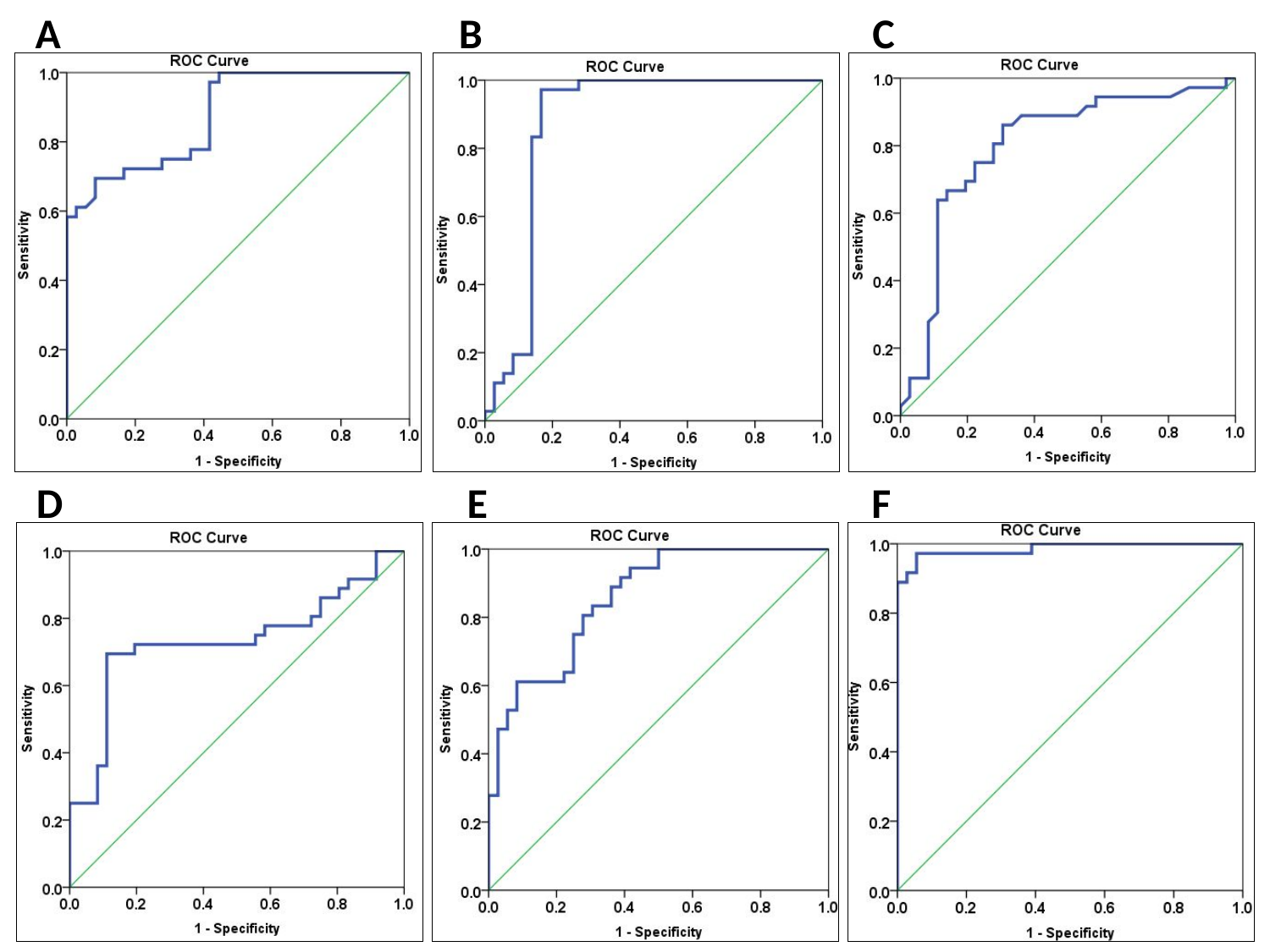

A
B
C
D
E
F
